# Time-gated Raman spectroscopy and proteomics analyses of hypoxic and normoxic renal carcinoma extracellular vesicles

**DOI:** 10.1038/s41598-021-99004-6

**Published:** 2021-10-01

**Authors:** Anatoliy Samoylenko, Martin Kögler, Artem Zhyvolozhnyi, Olha Makieieva, Geneviève Bart, Sampson S. Andoh, Matthieu Roussey, Seppo J. Vainio, Jussi Hiltunen

**Affiliations:** 1 Laboratory of Developmental Biology, Disease Networks Research Unit, Faculty of Biochemistry and Molecular Medicine, University of Oulu and Kvantum Institute, 90014 Oulu, Finland; 2 VTT Technical Research Centre of Finland, 90570 Oulu, Finland; 3 Institute of Photonics, University of Eastern Finland, 80101 Joensuu, Finland

**Keywords:** Proteins, Raman spectroscopy, Renal cell carcinoma, Renal cell carcinoma

## Abstract

Extracellular vesicles (EVs) represent a diverse group of small membrane-encapsulated particles involved in cell–cell communication, but the technologies to characterize EVs are still limited. Hypoxia is a typical condition in solid tumors, and cancer-derived EVs support tumor growth and invasion of tissues by tumor cells. We found that exposure of renal adenocarcinoma cells to hypoxia induced EV secretion and led to notable changes in the EV protein cargo in comparison to normoxia. Proteomics analysis showed overrepresentation of proteins involved in adhesion, such as integrins, in hypoxic EV samples. We further assessed the efficacy of time-gated Raman spectroscopy (TG-RS) and surface-enhanced time-gated Raman spectroscopy (TG-SERS) to characterize EVs. While the conventional continuous wave excitation Raman spectroscopy did not provide a notable signal, prominent signals were obtained with the TG-RS that were further enhanced in the TG-SERS. The Raman signal showed characteristic changes in the amide regions due to alteration in the chemical bonds of the EV proteins. The results illustrate that the TG-RS and the TG-SERS are promising label free technologies to study cellular impact of external stimuli, such as oxygen deficiency, on EV production, as well as differences arising from distinct EV purification protocols.

## Introduction

Of the kidney associated cancers the Renal Cell Carcinoma (RCC) represents the most common type, being among the top ten cancer forms worldwide^1^. Despite surgery and medication therapy, patients with RCC still have a poor prognosis. Lack of clinically applicable prognostic markers and the failure to eradicate RCC associated metastatic lesions are the main challenges facing RCC treatment^2^.

Recent advances in extracellular vesicle (EV) research have demonstrated that eukaryotic cells secrete spherical particles (0.03–1 μm diameter) that are enclosed by phospholipid bilayer^3^. These vesicles typically contain various proteins, RNA species and metabolites. EVs can be isolated from many body fluids such as blood, urine, sweat, saliva, cerebrospinal fluid and also from culture media of many cell types. There are evidences that the EV subgroup named exosomes (30–100 nm), which derive from the endosomal pathway and are released by multivesicular bodies, is involved in intercellular signaling, and seems important for primary tumor growth but also for formation of metastatic foci^4^. The discovery that EVs composition reflects the physiological status of a cell that produces EVs offers excellent grounds to develop novel diagnostic tools for early-stage cancer identification.

Rates of EV production by cells and composition of their molecular cargo are regulated by external stimuli such as hypoxia^5^. Intratumoral hypoxia that is caused by reduced oxygen supply is a key factor towards cancer initiation, progression, and formation of metastatic nodes^6^. As for the molecular pathways in hypoxia VHL (Von Hippel–Lindau) is an important tumor suppressor gene, that regulates stability of the hypoxia inducible factor (HIF) and whose mutations are present in around 70% of sporadic RCC^7^. EVs released by hypoxic cells have effects on many hallmarks of cancer such as cell survival, proliferation, angiogenesis, invasion and metastasis^5^ and these functions are connected to hypoxic EV molecular cargo, such as proteins and miRNAs^5^. EV cargo can be identified by proteomics and transcriptomics, but these methods require too much workload to be used in routine clinical diagnostics. Even though EVs are promising diagnostic and therapeutic liquid biopsy components, their wide use for such purpose is essentially requiring better methods for EV isolation and characterization.

Raman spectroscopy (RS) is a powerful method to optically characterize complex mixtures of biomolecules. It is based on inelastic scattering of photons by molecules. RS provides a unique “fingerprint” spectrum of biomolecules in a non-invasive and label free manner^8^. During the last decade, some Raman related studies have been performed to characterize biochemical profiles of EVs from different biological samples. These studies have mainly used either a label-free surface-enhanced Raman spectroscopy (SERS) or an indirect EV detection with SERS tags (reviewed by^9,10^). Both of these approaches convincingly demonstrated the potential of SERS for discrimination between cancerous and non-cancerous EVs.

The main advantage of RS and SERS in analysis of liquid samples is that the resulting spectra are not strongly affected by vibration of the water molecules^11^. However, sample induced fluorescence of many biological compounds is a limiting factor in studying EVs with RS. SERS can quench fluorescence, but only when metallic nanoparticles or patterned nanostructures are in close proximity, typically a few tens of nanometers, to the target molecules. This is challenging in liquid phase and the repeatability of results cannot always be ensured, since SERS enhancement and thus fluorescence quenching is distance dependent^12^. Fluorescence is especially problematic, when EVs are labelled with chemical fluorescent dyes or carry fluorescent proteins, commonly used to study EVs intracellular uptake and biodistribution^13^.

While the majority of SERS studies compared EVs isolated from cancerous versus “healthy” cell lines, we analyzed the changes caused by hypoxia treatment in the EVs released by cells of the same line. In this way, we aimed to reduce heterogeneity of EVs by avoiding cell line-specific features. The motivation was to investigate the usability of RS to reveal potential changes in EV structures. In the present study, we focused to analyze EVs with the time-gated Raman spectroscopy (TG-RS) and also combined this approach with SERS (TG-SERS) to improve further the signal-to-noise ratio. TG-RS increases specificity and intensity of the Raman signal while suppressing, i.e. “gating out” the sample derived autofluorescence. TG-RS has been applied earlier to biological samples^14–17^ (for more detailed information about the TG-RS see Kögler and Heilala^18^).

We show that hypoxia treatment induced production of EVs from RCC-derived Renca and 786-O cells and caused notable differences in the EV protein content as judged by Mass spectrometry (MS) and Western blot analysis. Hypoxia-induced EV proteins in Renca were mostly associated with plasma membranes and cell adhesion/integrin complexes. Among exosomal/EV markers CD9 expression was increased most prominently by hypoxia treatment. Studying EV samples with TG-RS and TG-SERS indicated that the Raman spectra can distinguish hypoxic EVs from those secreted by the RCC cells under normoxia. Interestingly, EVs isolated by using two different approaches (gradient ultracentrifugation and size-exclusion chromatography) depicted multiple RS spectral differences, and thus different EV subpopulations with associated membrane cargo and co-purified components^19^. We conclude that TG-RS offers a useful, label free and relatively fast method to characterize the composition of EV samples.

## Materials and methods

### Cell culture

Mouse renal adenocarcinoma-derived Renca cells (ATCC CRL-2947) and human renal adenocarcinoma-derived 786-O cells (300107, CLS Cell Lines Service, Eppelheim, Germany) were cultured in Dulbecco’s modified Eagle medium (DMEM)/F-12 (Gibco, 31331028), supplemented with 10% fetal bovine serum (FBS), 100 U/ml penicillin and 100 µg/ml streptomycin at 37 °C in 5% CO_2_. For experiments 5 × 10^6^ cells were plated per 15 cm dish (Greiner Cellstar, 639160). Cell viability was tested with Trypan Blue Stain (T10282 Invitrogen) in TC20 Automated Cell Counter (145-0101 Biorad) and was in the range of 95–97%. The whole-cell lysates were prepared by adding 2 ml of lysis buffer (RIPA buffer solution (R0278 Sigma-Aldrich) with proteinase (P5726 Sigma) and phosphatase (50892791001 Roche) inhibitors) per 15 cm dish. After 3 min. incubation at RT, cell lysates were transferred to Eppendorf tubes and further incubated on shacking platform for 30 min at + 4 °C. The proteins were pelleted by centrifugation at 15,000*g* for 20 min.

### EV purification

For the first 24 h after plating, Renca and 786-O cells were maintained in 15 cm cell culture dishes under normoxia (21% oxygen) until 80% confluence, after which medium was changed to medium without FBS (20 ml per dish) and cells were kept for the next 24 h either under normoxia or hypoxia (1% oxygen). After that, the medium was collected for EV isolation. EVs were purified from cell culture media (300 ml per condition) by one of the following two methods: combination of sequential ultracentrifugation and size-exclusion chromatography with Exo-spin columns (Cell Guidance Systems Ltd), or Optiprep-based gradient ultracentrifugation (Supplementary Figure S1). In brief, collected medium was centrifuged at 5000*g* for 15 min to remove floating cells and cell debris, and the obtained supernatant was concentrated using Centricon Plus-70 filter units (Merck Millipore, cut-off 100 K) for 10–20 min at 2500*g*.

For ultracentrifugation, concentrated samples were diluted with PBS to the total volume of 10 ml, centrifuged at 100,000*g*, 4 °C (Sorvall TH-641 rotor) for 15 h and pellets suspended in 200 µl PBS. Obtained suspensions were further purified using Exo-spin kit (EXO3) according to the manufacturer’s protocol. Each sample was eluted in 200 µl of PBS.

For gradient ultracentrifugation, 50% Optiprep (60% iodixanol; Sigma Aldrich): buffer A (0.25 M sucrose, 6 mM EDTA, 60 mM Tris–HCl, pH 7.4) mixture was diluted with buffer B (0.25 M sucrose, 1 mM EDTA, 10 mM Tris–HCl) to prepare 5%, 10%, 20%, 40% Optiprep gradient solutions. These solutions were loaded into the Beckman polymer tube layer by layer from the highest (bottom) to lowest (top) iodixanol concentrations (10 ml in total). Concentrated supernatants (prepared as described above) were loaded to the top of the tube. Samples were ultracentrifuged at 100,000*g*, 4 °C (Sorvall TH-641 rotor) for 15 h. Eleven fractions (1 ml each) were collected from the top to the bottom of the gradient and resuspended in 10 ml of PBS for the further 15 h ultracentrifugation at 100,000*g*, 4 °C (Sorvall TH-641 rotor). After supernatants removal, the pellets were resuspended in 200 µl of PBS. Each fraction was assayed by nanoparticle tracking analysis (NTA) and electron microscopy and the fractions with the highest concentration of EVs (fractions 5–7; density 1.07–1.12 g/ml) were combined and used for further experiments. Total protein yields, determined by Pierce™ BCA Protein Assay Kit (ThermoFisher Scientific, 23225), were 1.15 ± 0.30 µg (Renca normoxia ExoSpin), 1.53 ± 0.40 µg (Renca hypoxia ExoSpin), 0.17 ± 0.9 µg (Renca normoxia gradient), 0.185 ± 0.1 µg (Renca hypoxia gradient), 1.18 ± 0.46 µg (786-O normoxia ExoSpin), 1.43 ± 0.31 µg (786-O hypoxia ExoSpin) (calculated per million cells).

Further details of EV isolation and characterization are available in the EV-TRACK knowledgebase (EV-TRACK ID: EV210179)^20^.

### NanoSight measurements

The concentrations and size distributions of the EV samples were characterized by NTA using the Malvern Panalytical NanoSight NM300 instrument equipped with a 405 nm laser. Double distilled water was used to make 1:1000 dilutions before measurements. Four or eight 60 s videos were recorded of each sample with camera level 14 and detection threshold set up at three. Data were analyzed with NTA software version 3.4. Analysis of variance was performed using GraphPad Prism 8 software, with *p* ≤ 0.05 considered to be statistically significant.

### Electron microscopy

EV samples were analyzed by transmission electron microscopy (TEM). 2 µl of each sample were deposited on a Formvar carbonated grid (glow-discharged) and after negative staining with 2% uranyl acetate and immunostaining with anti-CD63 antibody (LAMP-3, MBL, Nagoya, Japan; 1:100 dilution) examined using the Tecnai G2 Spirit transmission electron microscope (FEI, Eindhoven, The Netherlands). Protein A-gold complex (10 nm) served to detect the primary anti-CD63 antibody. Images were captured with a charge-coupled device camera (Quemesa, Olympus Soft Imaging Solutions GMBH, Münster, Germany) at 1:49,000, 1:30,000, and 1:18,500 magnifications.

### Western blot analysis

Isolated EVs were lyzed in RIPA buffer (Cell Signaling Technology) containing protease inhibitor cocktail cOmplete ULTRA (Roche) and phosphatase inhibitor cocktail 2 (Sigma-Aldrich). Proteins (10 µg per sample) were separated on 10% SDS PAGE gel, and then transferred to nitrocellulose membrane. Antibodies against exosomal/EV markers CD81 (Santa Cruz Biotechnology, sc-166029), CD9 (Abcam, ab92726), ALIX (Abcam, ab117600), and TSG101 (Santa Cruz Biotechnology, sc-7964), as well as anti-GM130 (BD Biosciences, 610822) and anti-Argonaute-2 antibody (Abcam, ab32381) (all at 1:1000 dilutions) were incubated overnight at 4 °C with the membranes, and washed several times in PBST buffer. Total proteins on membranes were stained with Ponseau S and total stain Q (Azure Biosystems). The respective secondary peroxidase-conjugated IgG antibodies (Invitrogen) at 1:5000 dilutions were then applied to the membranes. The Lumi-Light Western Blotting Substrate (Roche Diagnostics, Switzerland) was used to visualize the bound antibodies.

### Analysis of EVs using ExoView

For analyzing expression of EV biomarkers and EV quantification the ExoView R100 platform (NanoView Biosciences, Boston) was used. In ExoView the anti-tetraspanins antibodies are immobilized on the chips and bind the EVs for analysis. At the next step of the assay, the chip-fixed EVs are stained with fluorescently labelled antibodies against specific EV proteins.

The mouse ExoView Tetraspanin (EV-TETRA-M2) and human ExoView Tetraspanin (EV-TETRA-C) kits were used. The samples were processed according to the manufacture’s protocol. Briefly, cell culture media were centrifuged at 5000*g* for 15 min to remove cell debris, after that normoxia and hypoxia samples (1 µg protein) were carefully loaded onto each chip and incubated for the 24 h. After that, the chips were washed three times on an orbital shaker to remove unbound particles. The chips were incubated for one hour with the human anti-CD81 (BD Pharmingen 555675), human anti-CD63 (BD Pharmingen 556019), human anti-CD9 (Biolegend V P018), mouse anti-CD81 (Biolegend 104902), mouse anti-CD63 (Biolegend 143902), mouse anti-CD9 (Biolegend 124802) fluorescently labelled antibodies. Mouse IgG (Biolegend 400101) and rat IgG (Biolegend 400502) were used as controls for human and mouse samples, correspondingly. The immunostained chips were washed three times in PBS, once in deionized water and dried. Image and data acquisition of the stained chips were performed with the ExoView R100 (NanoView Biosciences) and the data analysis with the ExoViewer 3 (NanoView Biosciences).

### Raman measurements and analysis

EVs were analyzed with a wavelength calibrated commercial time-gated Raman spectrometer (PicoRaman from Timegate Instruments, Oulu, Finland), using pulsed laser λ_exc_ = 532 nm excitation at 100 mW of laser power with 100 ps pulse length at around 100 kHz repetition rate, within 2 weeks after samples isolation. The TG-RS system encompassed a temperature stabilized CMOS SPAD array (8 × 768 pixels single photon counting) detector with spectral resolution of 5 cm^−1^ and temporal resolution of 100 ps. The TG-RS was coupled to a conventional non-immersion Raman probe with quartz window material (BWTek, BAC102, Metrohm, Herisau, Switzerland) and a working distance of 5.4 mm. Reference analysis was performed with continuous wave (CW) laser excitation at λ_exc_ = 514 nm, using a confocal Raman microscope (InVia from Renishaw, Wotton-under-Edge, UK) with cooled CCD detector and 0.3 cm^−1^ spectral resolution. A maximum of 5 mW of CW excited laser emission was guided through a lens (Leica, N Plan, Wetzlar, Germany) with a magnification factor of 20× and numerical aperture (NA) of 0.4, while the spectral acquisition time to the samples was set to 10 s. In comparison, the overall measurement time for each sample with TG-RS/SERS was set to be 3 times 60 s including repetitions to achieve an appropriate signal without sample evaporation. For performing RS measurements small aluminum microwells with a cavity in µL scale were used, as previously shown^14^. This allows to first measure the Raman response of isolated EV samples while subsequently measure the SERS response. Aluminum does not interfere with the compounds of interest in the fingerprint region i.e. 400–1800 cm^–1^^50^ and thus is an easily available and perfect substrate also to investigate EVs optically. Approximately 5 µL EVs sample volume was pipetted onto an aluminum cavity and TG-RS as well as CW Raman spectra were recorded. Following the Raman measurements, SERS measurements were conducted. For SERS, commercial silver nanoparticle (Ag NPs) solution with a 40 nm particle size (Ag NP; #730807) purchased from Merck Sigma-Aldrich (Darmstadt, Germany) was used. The Ag NP stock solution was centrifuged with MIKRO 120 centrifuge (Hettich, Tuttlingen, Germany) at 2800*g* for 5 min., followed by the removal of supernatant, reaching the final concentration of about 0.06 g L^−1^.

TG-RS data was post-processed with Timegate Instruments software. Spectral data analysis was performed with OriginPro (V. 2020a and 2020b, OriginLab, Northampton, MA, USA). All data was intensity normalized within an interval of 0 and 1 and plotted with an offset for better presentation.

### Proteomics

Proteins were extracted from individual EV samples by using methanol/chloroform precipitation. Dried protein pellets were diluted in 4× Laemly loading buffer containing 10% β-mercaptoethanol, loaded into 12% SDS polyacrylamide gel (PAAG) (12% Mini-PROTEAN TGX Precast Protein Gel, Bio-Rad) and run for maximum 15 min at 100–110 V. SDS gel pieces stained with Sypro Ruby (Sigma, S4942) were cut out and processed as follows: 3 × 5 min washing steps with 50 mM ammonium-bicarbonate in 40% acetonitrile/60% water to destain the gel, reduction with 20 mM DTT for 30 min at room temperature, alkylation with 45 mM iodoacetamide for 30 min at room temperature, washing and tryptic digestion with 5 μl of trypsin solution (20 ng/μl proteomics grade trypsin (Sigma) in trypsin buffer (40 mM ammonium-bicarbonate in 9% acetonitrile/91% water)) overnight at 37ºC. The supernatant was transferred to a sample vial before the gel piece was extracted a second time with 15 μl of 0.1% trifluoroacetic acid (TFA) in water. The combined extracts were centrifuged and 25 μl of the supernatant were transferred to a sample vial to allow LC–MS (Liquid chromatography–mass spectrometry) analysis using an Easy-nLC 1000 (Thermo Scientific) system coupled to a Fusion Lumos Tribrid mass spectrometer (Thermo Scientific). Peptides were trapped on an AcclaimPepmap 100 C18 3 µm, 0.075 × 2 mm (Thermo Scientific) trap column and separated on a Thermo AcclaimPepmap RSLC C18 2 µm, 0.075 × 150 mm analytical column, using a gradient from 97% A (0.1% formic acid) to 35% B (0.1% formic acid in CAN) over 90 min, flow 0.3 µl/min. The mass spectrometer was operated in 3 s cycles where the MS spectra were recorded with the orbitrap analyzer at resolution 120,000 allowing the collection of up to 4e5 ions for maximal 50 ms before switching to MSMS mode. Multicharged ions (threshold 5e4) were fragmentated with equal priority by HCD (30% collision energy) and CID (35% collision energy, 10 ms activation, Q 0.25) using quadrupole isolation with 1.6 Da width and 21 s dynamic exclusion. HCD ions (up to 5e4 ions) were collected for max 200 ms in the orbitrap analyzer at a resolution of 15,000. Collision-induced dissociation (CID) ions were recorded in the ion trap (rapid mode) aiming at higher sensitivity (threshold 1e4).

Raw data were processed and analyzed with Proteome Discoverer (Thermo Scientific version 2.2) using Sequest as search engine. MSMS spectra recorded in the ion trap were processed with 0.6 Da mass tolerance, orbitrap data with 0.02 Da. Raw data were recalibrated with the mouse Swissport database (version 2017-10-25) and searched with the following settings: precursor mass tolerance 10 ppm, trypsin cleavage with up to 2 missed cleavages, carbabmidomethyl as fixed modification on cysteine, oxidation as optional modification on methionine, deamidation optional on Gln and Asn, protein N-terminus optionally acetylated. The percolator node was used with FDR 0.01.

The minora feature detector was activated for label free quantification using 2 min RT alignment and precursor intensity normalized to total peptide amount. Peptide amounts were calculated from precursor intensities (area based) and normalized to total peptide amounts. Protein hits with more than 1 peptide/protein were accepted, minimum replicate features were set to 50%.

Functional classification of proteins was performed using the Gene Ontology (GO) knowledgebase (http://geneontology.org/). PANTHER (Protein ANalysis THrough Evolutionary Relationships) Overrepresentation Test (Released 20200728) was performed using GO Ontology database (https://doi.org/10.5281/zenodo.4081749 Released 2020-10-09). Statistical analysis was done with Fisher’s exact test using the Bonferroni correction for multiple testing. GO annotations with Bonferroni-corrected *p* < 0.05 and enrichment fold ≥ 5 were visualized in scatterplots with REViGO (http://revigo.irb.hr/)^21^. Venn diagrams were prepared using Venny (version 2.1) (http://bioinfogp.cnb.csic.es/tools/venny/).

## Results

### Characterization of EVs secreted by RCC cells

**Figure 1:**
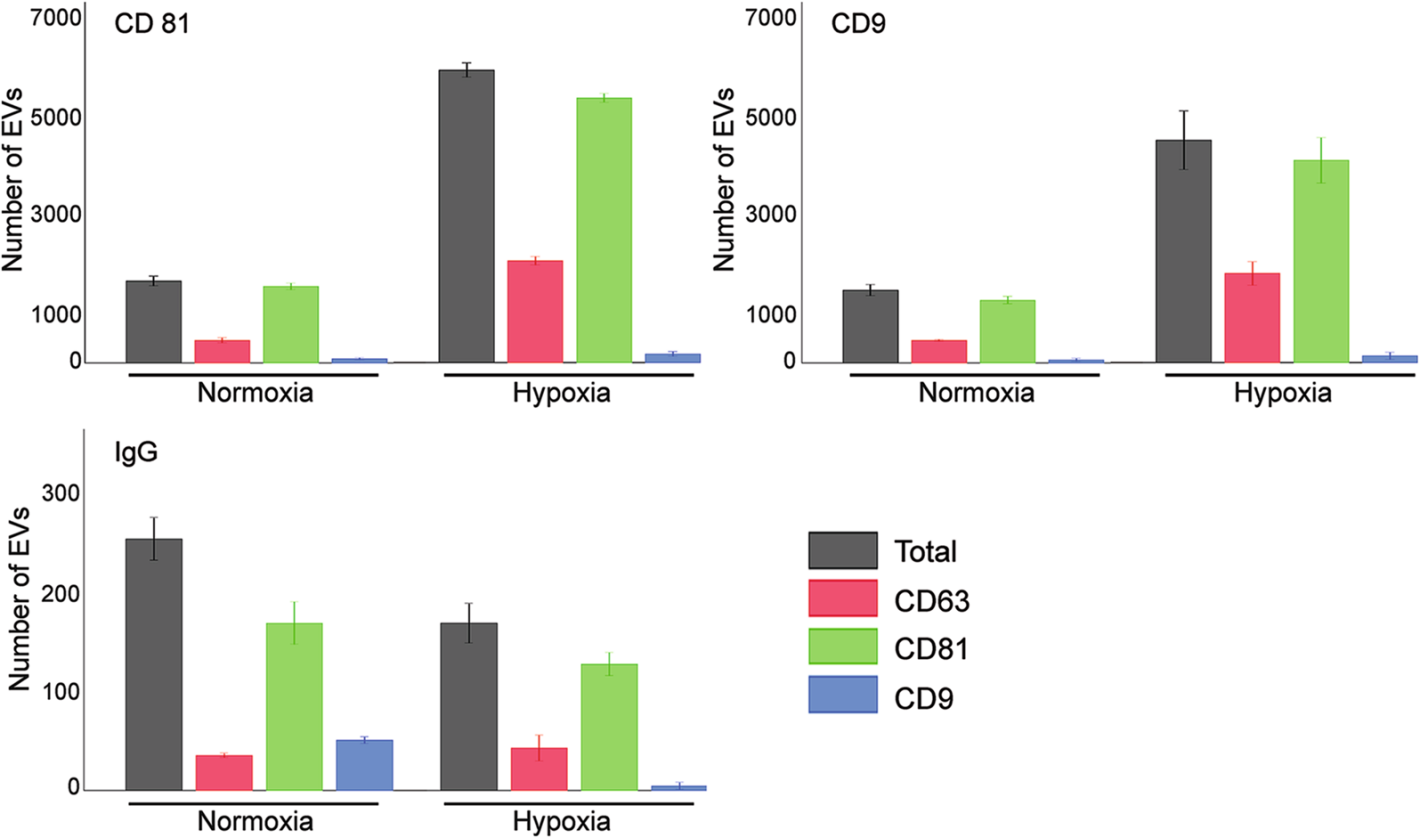
Characterization of EVs produced by Renca cells cultured under normoxia or hypoxia by ExoView. Names of the capture chips are shown in the upper left corners, total number of detected EVs in a sample (1 µg total protein) are shown by grey bars, number of EVs expressing CD63, CD81, and CD9 depicted by red, green, and blue bars, correspondingly.

To study to what extend hypoxia may impact secretion of EVs by the Renal Cell Carcinoma (RCC)-derived Renca cells, these cells were cultured in FBS-free medium for 24 h under normoxic (21% oxygen) or hypoxic (1% oxygen) conditions. The amount of EVs expressing typical exosomal markers CD9 and CD81 was analyzed in debris-free cell culture supernatants by using ExoView platform. We found that number of EVs captured on chips functionalized with either CD9 or CD81 antibodies in supernatants form cells cultured under hypoxia was 3.1–3.6 times higher as compared to normoxia (Fig. 1). By contrast, the number of EVs non-specifically bound to control IgG chip in hypoxic sample was even lower as compared to normoxic one. Relative distribution of exosomal markers did not change between the conditions, with majority of EVs were CD81 positive, about 1/3 CD63 positive, while minority expressed CD9. No differences in mean size between EVs from hypoxia and normoxia samples (63 ± 23 nm at normoxia and 63 ± 15 nm at hypoxia for CD81 probe, 57 ± 12 nm at normoxia and 61 ± 17 nm at hypoxia for CD9 probe) were noted.

EVs were purified from Renca cell culture medium by density gradient ultracentrifugation or alternatively by ultracentrifugation followed by size exclusion chromatography with Exo-spin columns (Cell Guidance Systems Ltd) (Supplementary Figure S1). Altogether, six independent EV isolations using Exo-spin and three EV isolations with density gradient for each condition (hypoxia or normoxia) were conducted and the obtained EVs analyzed.

**Figure 2:**
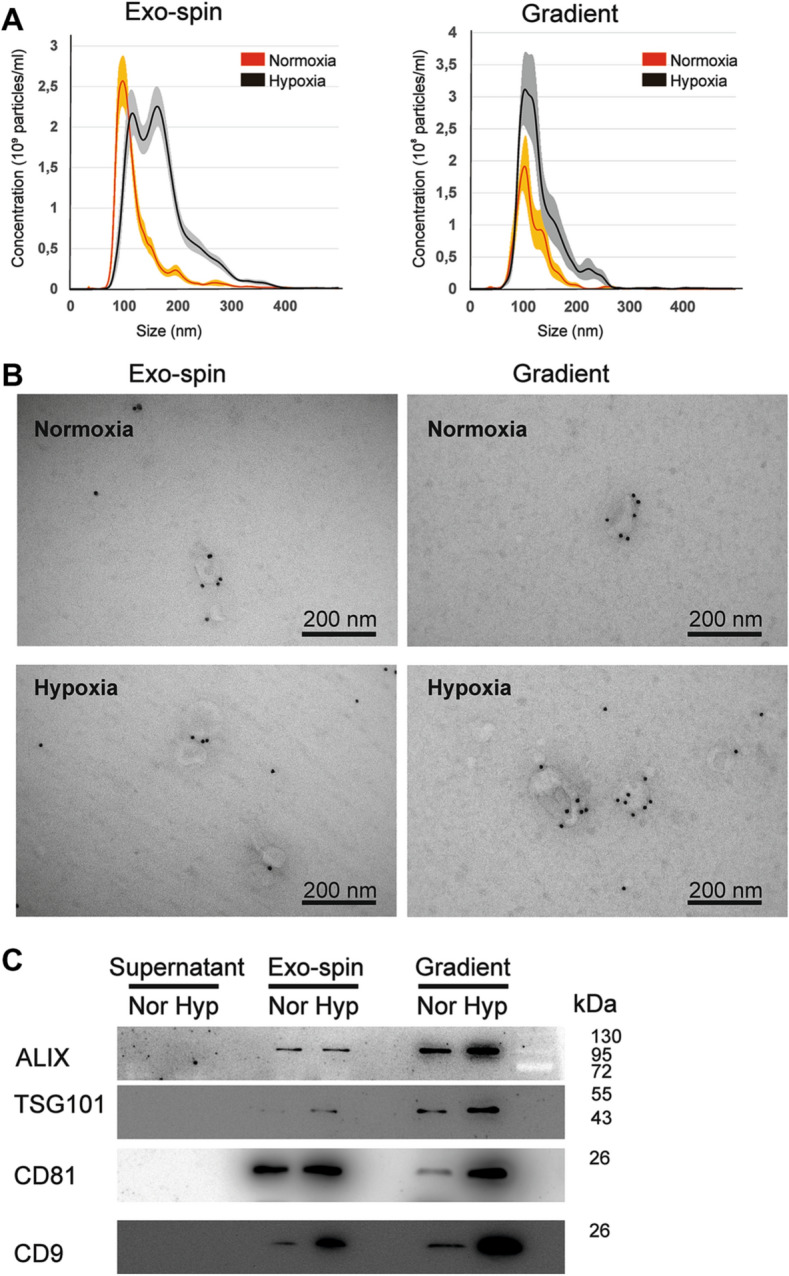
Characterization of EVs produced by Renca cells cultured under normoxia (Nor) or hypoxia (Hyp) and isolated by using either density gradient ultracentrifugation (Gradient) or sequential ultracentrifugation followed by Exo-spin size exchange chromatography (Exo-spin). (**A**) EV concentrations and size distribution measured by NTA. (**B**) Immuno-TEM with anti-CD63 antibody (magnification 1:49,000, images of the same regions with lower magnification are shown in Supplementary Figure S2A). (**C**) Western blot with antibodies against common EV markers (images of uncropped blots are shown in Supplementary Figure S2D).

Number and size distribution of EVs were characterized by nanoparticle tracking analysis (NTA). In Exo-spin-purified samples, EVs released by cells growing at hypoxic conditions displayed broader range of sizes as compared to normoxia, with mean EV sizes of 167.8 ± 4.3 nm and 130.9 ± 3.6 nm, correspondingly (Fig. 2A). Hypoxic EVs depicted two distinct size peaks (at 117 nm and 163 nm), while normoxic EVs provided one (at 99 nm).

Density gradient-based isolation reduced the number of EVs of bigger sizes in RCCs exposed to hypoxia, therefore the second peak of EVs disappeared and the size distributions appeared more similar to normoxia (mean sizes 127.7 ± 4.1 nm in hypoxia as compared to 108.4 ± 4.9 nm in normoxia). The amounts of EVs in hypoxic samples were higher than in normoxic ones independently of the isolation method used (2.60e + 11 ± 2.28e+10 particles/ml versus 1.36e+11 ± 1.13e+10 particles/ml for Exo-spin, *p* ≤ 0.01 in a two-tailed *t* test; 2.10e+10 ± 4.20e+09 particles/ml versus 9.96 +09 ± 1.87e+09 particles/ml for density gradient; *p* ≤ 0.01 in a two-tailed t test).

Analysis of isolated RCC-derived EVs by transmission electron microscopy (TEM) (Tecnai G2 electron microscope) with negative staining only or immunolabeling with anti-CD63 antibody (Fig. 2B; Supplementary Figure S2) revealed EVs with size range from 30 to 200 nm. Except for the smaller EVs, not detectable by NTA, the observed size distributions shown by the TEM analysis were in accordance with NTA data. The majority of detectable EVs expressed CD63, representing a widely used exosomal/EV biomarker^22^.

Western blotting was used to analyze more in depth the RCC EVs with antibodies against ALIX, TSG101, CD81 and CD9 (Fig. 2C)^3^. An equal amount of proteins was loaded (Supplementary Figure S3A and B; Supplementary Figure S4A). Argonaute2 (Ago2) binding to free *miRNA* in serum^23^, and GM130, a Golgi membrane component, served as a negative blotting control (Supplementary Figure S3C and Supplementary Figure S4B). Indeed, these markers illustrated that the hypoxic condition induced robust EV secretion by the RCCs. This induction was more prominent in EV samples isolated by density gradient centrifugation. Those EVs that expressed the CD9 marker were most notably induced by hypoxia (Fig. 2C).

In addition to mouse RCC we also analyzed the effect of hypoxia on EV production by 786-O cells, derived from human RCC. Analysis of cell culture supernatants with ExoView showed that hypoxia induced production of EVs by 786-O cells, though to a much lower extend as compared to mouse RCC (Supplementary Figure S5). After EVs were isolated from 786-O cell culture supernatants by using Exo-spin (representative TEM images shown in Supplementary Figure S6B), higher amount of EVs in hypoxia samples was demonstrated by NTA as well (1.09e+11 ± 9.83e+9 particles/ml versus 5.61e+10 ± 3.57e+9 particles/ml, *p* ≤ 0.01 in a two-tailed *t* test; Supplementary Figure S6A).

Unlike mouse Renca, in 786-O cells no relevant changes in EV size were observed (mean sizes 153.5 ± 3.5 nm and 177.5 ± 8.7 nm for hypoxia and normoxia, correspondingly). Induction of CD81-positive EVs under hypoxia was observed in Western blot (Supplementary Figure S7).

We conclude that hypoxia stimulates production of a wide range of EVs from the RCC cells, and the density gradient based purification results in a more homogeneous EV population enriched in EV/exosomal markers, as compared to size exclusion chromatography approach.

### Identification of characteristic compounds with TG-RS

**Figure 3.**
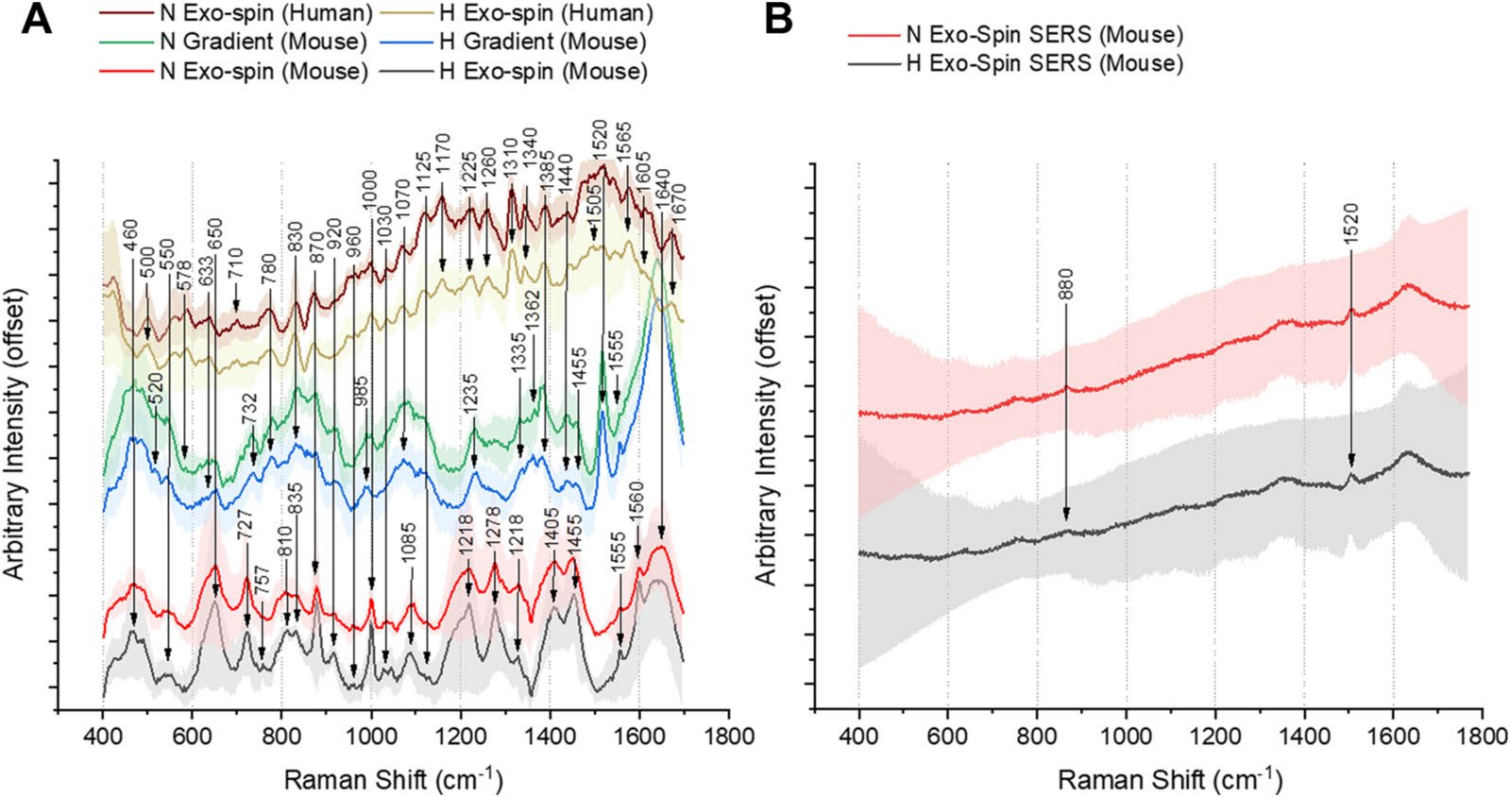
Influence of background fluorescence on RS measurements. The difference between mouse and human RCC with different spectrometer setups (**A**): TG-RS average spectra (λ_exc_ = 532 nm) of EVs isolated with density gradient (green and blue) and Exo-spin (red, black, brown, beige) and (**B**) conventional continuous wave excitation Raman (λ_exc_ = 514 nm) on average spectra of mouse RCC for normoxia (N—red) and hypoxia (H—black) EV samples.

The noted changes in the amount and molecular characteristics of EVs caused by hypoxia in the RCC model cancer cells provided a good way to assay whether Raman based spectral analysis would offer a useful high throughput EV analytic tool. Figure 3 exemplifies the difference in suppression of induced fluorescence background between TG-RS (Fig. 3A) and conventional CW Raman settings (Fig. 3B) using comparable excitation wavelength on hypoxic and normoxic samples. Whereas TG-RS allows to determine differences between normoxia (red, green and brown curves for mouse and human RCC) and hypoxia (black, blue and beige curves) EVs, with CW Raman these spectral features were not distinguishable. This is clearly observable for example within the amide III region (around 1235 cm^−1^). Prominent Raman bands and their origin shown in Fig. 3 are listed in Table 1. Supplementary Figure S8 shows TG-RS and CW Raman spectra of cell culture media samples after EV removal with 100,000*g* centrifugation (as controls).

**Table 1:** Tentative band assignment of Raman/SERS.

CW Raman	TG-Raman	Tentative Raman band assignments	Origin/category	References
440	460	ν(C–S)	–	^57,58^
	550–560	S–S stretching	Phosphates or cholesterol	^27^
	578	ν(S–S)	–	^57^
	624	C–C twisting mode	Aromatic ring deformation	^57^
640	655–666	ν(C–S) or C–C twisting mode of tyrosine	Tyrosine or Tryptophan	^27^
	710–731	DNA/CH_2_ rocking	(cAMP) or Adenine	^14,15^
750	757	Tryptophan	Tryptophan	^59^
	775–780	Amide IV (tryptophan)	Amide IV (tryptophan)	^25^
	810	O–P–O stretch	RNA or phosphates	^28^
	830	Tyrosine	Tyrosine	^24^
	850	C–C ring breathing mode in tyrosine or polysaccharide structure	Tyrosine or media compounds	^25,59^
870–880	877–880	Ethanol or acetate in media	Ethanol or acetate	Reference measurements, this work
	920	N–C_α_–C stretch	Glucose or lactic acid	^14,15^
	960	C–C skeletal stretch in protein (β-sheet)	CH bend	^25,28^
	990–995	Uracil or 12-methyl-tetradecanoic acid	–	^29^
	1000	R breathing	Phenylalanine	^15,27^
	1030–1040	ν(C–N)	Glycogen or proline	^25,59^
	1050–1070	C–O and C–N stretching of proteins	Proteins	^59^
1080	1085	Ethanol	Ethanol	Reference measurements, this work
	1120–1130	C–N stretch in polypeptide chains	β-d-glucose	^29^
	1170–1180	C − O − C or P − O stretch	Phosphates, tryptophan or tyrosine	^14,28^
	1218–1221	ν(C − C)	Tyrosine, Phenylalanine	^57^
	1235	CONH group	Amide III	^25^
1277	1278	Ethanol	Ethanol or amide III	Reference measurements, this work
	1310	CH_2_ twist	Lipids	^28^
	1330–1335	CH_3_CH_2_ wagging mode	Polynucleotide chain (purine bases)	^59^
	1350–1362	Ferri (Fe^3+^)	hemoproteins, Nucleotide	^14,52^
	1385	Aromatic ring vibrations of nucleic acids	DNA/RNA macromolecules	^60^
	1405	δ(CH_3_), ν(COO^−^)	-	^57^
	1440	C − H defect	Nucleobase	^28^
1455	1455	C − H deformation (CH_2_)	Lipids or cholesterol	^25^
	1460	Deformation of hydrocarbon chains or ethanol	Ethanol	^60^, reference measurements, this work
	1494	Spermine	Spermine phosphate hexahydrate	^61^
1505–1514	1510	ν(R,r), ν(C − H)	Tryptophan	^27^
	1520	N–H bend and C–N stretch	Amide II, carotenoids	^59^
	1555–1560	Tryptophan: ν(R)	Amide II, Tryptophan	^27^
	1601–1610	Aromatic amino acids	Phenylalanine or Tyrosine	^15^
	1620–1625	Amino acids, Ferri (Fe^3^^+^)	hemoproteins	^25,52^
1640	1650	C = O stretch	Amide I	^25^

Figure 3A illustrates that TG-RS average spectra of EVs derived from six Renca Exo-spin, three Renca density gradient isolations, and three 786-O Exo-spin isolations result in characteristic differences that correlate with hypoxia and normoxia exposures. Similarities of spectral peaks for all isolations such as proteins signatures (tyrosine/tryptophan at 830 cm^−1^, phenylalanine at 1000 cm^−1^, 1220–1235 cm^−1^ and around 1600 cm^−1^) are visible in the amide (I to IV) regions^24^. Interestingly, the dominance of amide I is pronounced less for EVs produced by 786-O cells than for the Renca-derived EVs, probably reflecting differences in concentrations of EVs produced by these cell lines. Another differences between the cell lines are the missing C–H deformation (lipids or cholesterol cf. Table 1) around 1455 cm^−1^ and the distinct presence of C–O–C/P–O stretch in human RCC. Remnants of the EV purification procedure such as ethanol (a component of the buffer, in which Exo-spin™ columns were supplied; 880 and 1278 cm^−1^) are noticeable with CW Raman being projected better with TG-RS, cf. Fig. 3. Glucose (around 920 cm^−1^ and 1130 cm^−1^) and glycogen (1030 cm^−1^) are visible only with the TG-RS derived spectral data^25^.

The Raman spectra also illustrate differential patterns based on the selected EV purification methods. The dissimilarity of the 1520 cm^−1^ peak, assigned to tryptophan or carotenoid fragments, is noticeable for EVs that were purified via the density gradient, but not noted in the Exo-spin EV samples. This difference may be due to the changes in the amount of heme-containing proteins^26^. Well distinguishable peaks at 650–660 cm^−1^, whose quantities differed in the Exo-spin and the density gradient purified EV samples, are associated with phosphates, that are present in the dilution buffer and the culture media^27^.

The EV sample differences could also be determined by the shape, displacement and form of the Raman peaks, especially in the range of 780–960 cm^−1^ depicting the nucleic acids^28^. In the density gradient isolations, phenylalanine falls in to a spectral area with two prominent peaks in range of 990–995 cm^−1^ assigned typically to uracil^29^.

**Figure 4.**
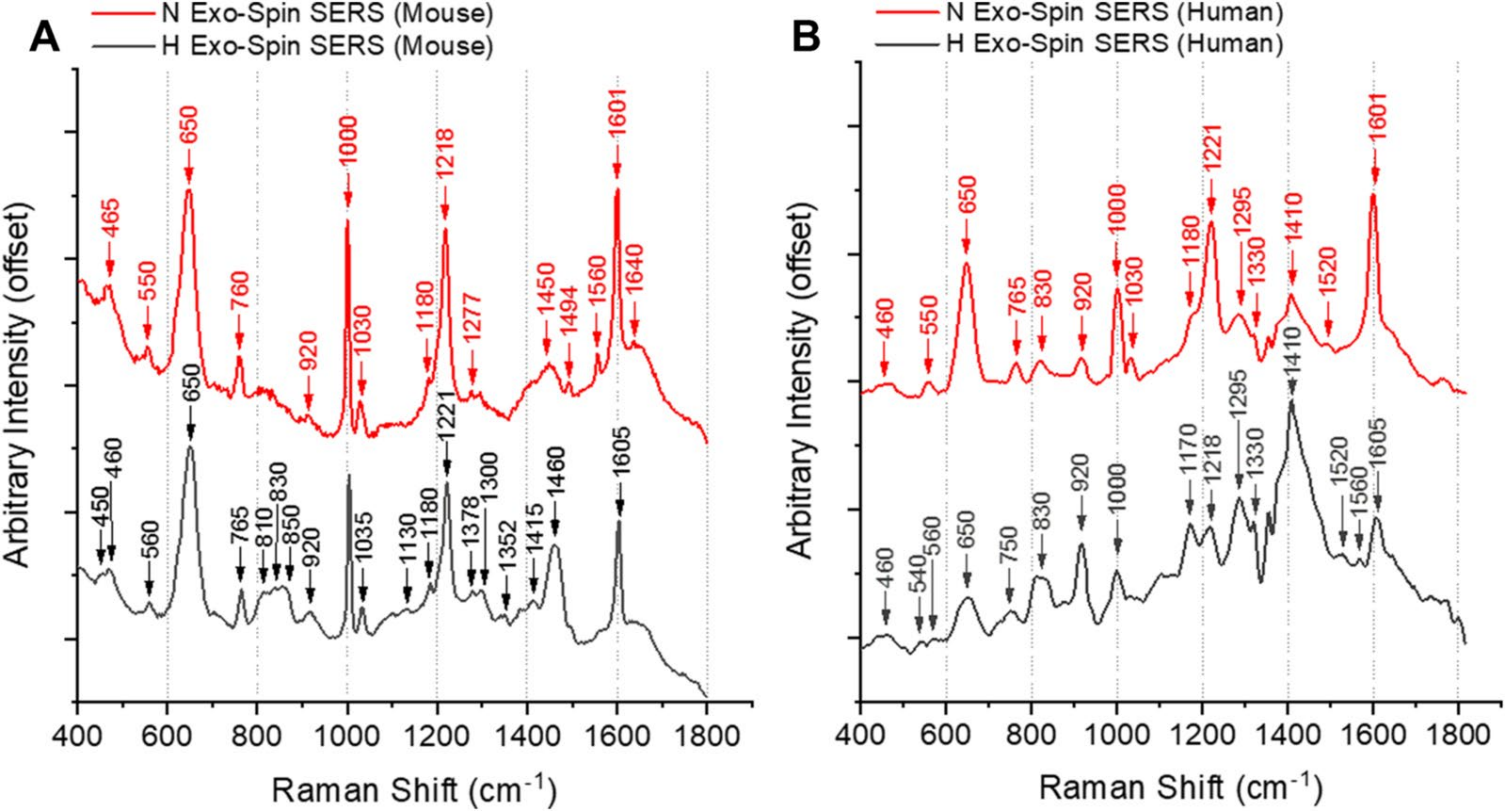
Comparison of EVs released by (**A**) mouse and (**B**) human RCC cultured under normoxia (N, red) and hypoxia (H, black) with TG-SERS (all samples isolated with Exo-spin).

The TG-RS detection can be further modified with the Ag NPs to boost the Raman signal resulting in a TG-SERS (see Fig. 4). Dominant spectral peaks at 650, 1000, 1220 and 1600 cm^−1^ are typically associated with proteins in biological samples. Since applied Ag NPs cannot cross the EV lipid membrane, the Ag NPs may enhance the optical field at the EV surface leading to more prominent spectra of the EV surface associated components. The other noted TG-SERS peaks appear to be at equivalent characteristics as depicted in TG-RS (compare Fig. 3A with Fig. 4A) mainly matching mouse and human RCC, but appearance of the peak around 1410 cm^−1^ can only be explained by higher DNA amount in the human RCC samples^15^. Also in human RCC the hypoxic condition can be differentiated by lower vibration of the C–C bond and the absence of the C–N bond (cf. Fig. 4B; Table 1). In general, as compared to the results of gradient isolations, Exo-Spin TG-RS and TG-SERS spectra show a few sharper and more distinguishable peaks. TG-SERS provides a clearer view and allows for better differentiation between hypoxia and normoxia. The large variation in spectral results appears to be the downside when enhancing the EVs Raman signal with Ag NPs, as can be seen in Supplementary Figure S9. Generally, enhancing Raman signal by SERS is well known to be vulnerable to repeatability issues, that are due to distance variations of the nanoparticles to the biomolecules of interest, thus it requires a uniform enhancement substrate^12^.

We conclude that TG-RS and TG-SERS, but not the conventional continuous wave excitation RS, provide useful approaches to analyse stimuli induced changes in the overall EV molecular composition as supported by the comparison of the Raman spectral data between the EVs purified from RCC cells exposed to hypoxia or normoxia. The RS approach seems also to be useful in illustrating the quality of EVs purified by different methods and cell lines with clear distinction between conditions of vesicles.

### Characterization of EV proteins by mass spectrometry

To complement Raman analysis and to study whether the differences in protein composition between EVs, released under hypoxia or normoxia, can be depicted with other approaches applied for the EVs analysis we took use of the mass spectrometry. We performed proteomics analysis of five Renca hypoxia/normoxia pairs of EV samples that were isolated with the Exo-spin columns. Each of these hypoxia or normoxia samples was analyzed in duplicates.

The proteomics analysis revealed in total 1388 unique high confidence proteins in the individual EV samples (Fig. 4A; Supplementary Table S1). Many markers that are characteristic for the exosomes and EVs such as CD81, CD9, CD63 and TSG101 were identified. These marker proteins were not present in the EV-depleted supernatants irrespective whether the cells were cultured under hypoxia or normoxia (Supplementary Table S2), in line with the Western blot results (Fig. 2C). Gene Ontology (GO) analysis (http://geneontology.org/) illustrated 60 biological processes that were significantly (*p* ≤ 0.05) overrepresented for the Renca EV proteins, and from them the inosine monophosphate (IMP) biosynthetic and metabolic processes appeared to be most prominently enriched (Supplementary Table S3).

**Figure 5.**
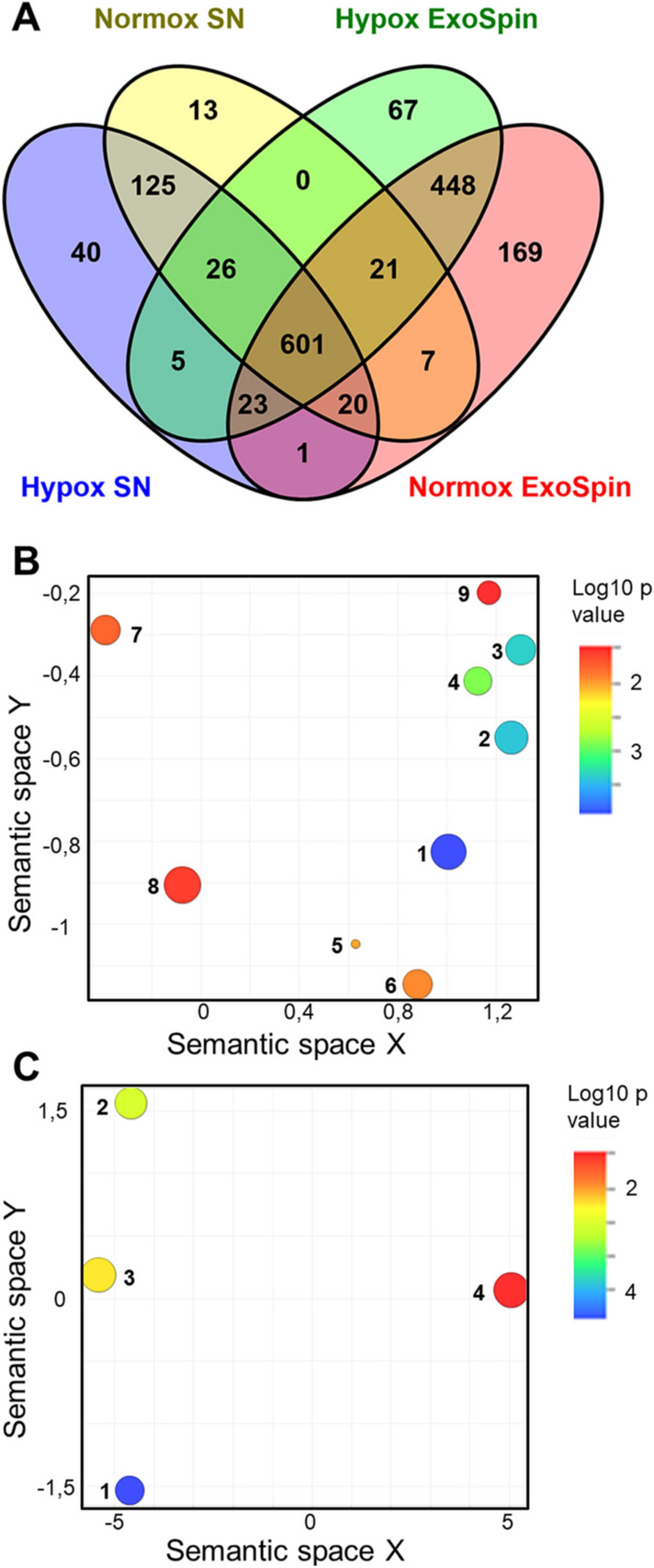
Proteomics analysis of EVs released under hypoxia and normoxia. (**A**) Comparison of proteins found in Exo-spin EV samples (hypoxia and normoxia) and proteins found in EV-depleted supernatants (SN) (hypoxia and normoxia). Actual protein lists are given in Supplementary Tables S1 and S2. (**B**) GO analysis of proteins significantly different in abundance between hypoxic and normoxic EVs for “Cellular components” and (**C**) GO analysis of the same proteins for “Molecular function”. GO Cellular component: 1. Plasma membrane protein complex; 2. Plasma membrane region; 3. Basolateral plasma membrane; 4. Lateral plasma membrane; 5. Integrin alpha5-beta1 complex; 6. Integrin complex; 7. Protein complex involved in cell adhesion; 8. Membrane protein complex; 9. Invadopodium membrane. GO Molecular function: 1. Integrin binding; 2. Cell adhesion molecule binding; 3. Receptor binding; 4. Macromolecular complex binding.

Of the 882 soluble proteins detected in EV-depleted supernatants we found that 704 were shared with the EV proteome (Fig. 5A). GO analysis showed 58 biological processes significantly (*p* ≤ 0.05) enriched in the soluble proteins secreted by Renca (Supplementary Table S4). Only 16 of the biological processes identified were simultaneously enriched for insoluble (EVs) and soluble (EV-depleted supernatants) proteins.

Comparison between hypoxic and normoxic EVs showed that a majority of identified proteins (1093, or 79%) were found in both normoxia and hypoxia groups, while 197 proteins were observed only in normoxic and 98 proteins in hypoxic samples (Supplementary Tables S5 and S6). In addition, analysis of peptide abundances identified that 29 proteins were noted both in hypoxic and normoxic samples, but differed significantly in their amounts (abundance ratio hypoxia/normoxia, adjusted *p* value ≤ 0.05) (Table 2). From these proteins, 26 were enriched in hypoxic samples, and the rest in normoxic. One of those proteins enhanced in expression under hypoxia was an exosomal/EV marker CD9. This was identified by the Western blotting as well (Fig. 2C). GO enrichment analysis for “cellular components” illustrated that the identified hypoxia-induced EV proteins were mostly associated with plasma membranes and cell adhesion/integrin complexes (Fig. 5B). GO analysis for “molecular functions” showed enrichment for cell adhesion/integrin binding as well, and also for receptor binding (Fig. 5C). The “biological process” category enriched for hypoxic EV proteins (*p* = 0.0132) was import of cargo into a target cell.

**Table 2:** Proteins significantly different in abundance between hypoxic and normoxic EVs.

Accession	Description	Abundance ratio: hyp/norm	Abundance ratio Adj. *p*-value
Q9Z1P8	Angiopoietin-related protein 4	0.037	0.002328
Q8R1L8	Angiopoietin-like protein 8	0.076	0.026312
P98063	Bone morphogenetic protein 1	0.086	0.03721
P35279	Ras-related protein Rab-6A	5.114	0.032794
Q9CQI3	Glia maturation factor beta	6.491	0.040723
Q9DAS9	Guanine nucleotide-binding protein g(i)/g(s)/g(o) subunit gamma-12	6.843	0.008032
P63044	Vesicle-associated membrane protein 2	7.432	0.008003
Q64735-1	Complement component receptor 1-like protein	7.795	0.030157
P35278	Ras-related protein Rab-5C	8.976	0.04162
Q3U9N9-1	Monocarboxylate transporter 10	9.483	0.002105
Q9Z0G9	Claudin-3	9.713	0.045659
P40240	CD9 antigen	9.805	0.046305
Q9R1Q7	Proteolipid protein 2	10.053	0.007324
Q8BGA2	LHFPL tetraspan subfamily member 2 protein	10.091	0.002328
Q8VDN2	Sodium/potassium-transporting ATPase subunit alpha-1	10.424	0.038954
Q62470	Integrin alpha-3	10.694	0.036181
Q9EPT5-1	Solute carrier organic anion transporter family member 2A1	10.934	0.033932
P10639	Thioredoxin	11.458	0.029611
P18572-1	Basigin	12.339	0.023675
P10852-2	Isoform 2 of 4F2 cell-surface antigen heavy chain	13.091	0.019798
P53986	Monocarboxylate transporter 1	14.121	0.015526
Q9Z127	Large neutral amino acids transporter small subunit 1	16.012	0.010218
P09055	Integrin beta-1	17.335	0.00784
Q99LX0	Protein/nucleic acid deglycase DJ-1	17.682	0.007324
O35566	CD151 antigen	17.939	0.006981
P11688	Integrin alpha-5	18.084	0.006795
Q8R3G9	Tetraspanin-8	18.9	0.005814
P51912	Neutral amino acid transporter B(0)	18.985	0.005573
P14094	Sodium/potassium-transporting ATPase subunit beta-1	19.265	0.005442

We conclude that EVs released by cells cultured under hypoxia are enriched with membrane-bound proteins known to participate in cellular processes such as adhesion and receptor binding.

## Discussion

Hypoxia (reduced oxygen concentrations), being a common feature of solid tumors, is associated with disease progression and poor prognosis in many types of solid cancers^30^. Hypoxic stress leads to multiple changes both inside tumor tissue and in tumor microenvironment, causing better adaptation of cancer to low oxygen levels. Changes in EVs production rates and composition are supposed to be one of the mechanisms by which cancer cells respond to hypoxic stimulus (reviewed by^31–33^).

Induction of EVs secretion by hypoxia, observed in our study, is a common phenomenon, shown, among other cell types, for melanoma, glioblastoma, prostate, ovarian, breast, and lung cancer-derived cells. Also non-cancer cells like cardiomyocytes^34^ and renal proximal tubular cells^35^ increase EV production upon hypoxic treatment. We found that hypoxia-induced EV production by mouse Renca cells was much stronger as compared to human 786-O cell, which may be related to the differences in HIF signaling in these two cell lines. Indeed, Renca cells express wild type VHL, while 786-O cells are VHL-defective and lack HIF-1α^36^.

Most studies found no differences in EV size distributions between hypoxic and normoxic EVs, though changes in EVs average sizes for different subfractions of EVs was reported for pancreatic cancer cell lines^37^. In our study, we found that a broader range of EVs (by size) is produced by RCC cells cultured under hypoxia as compared to normoxia when quantified by NTA, but did not detect such differences in sizes using the ExoView based analysis. These discrepancies may be due to the different optimal detection ranges for ExoView (50–100 nm) and NTA (above 90 nm)^38^.

Several publications described differences in protein composition between EVs produced under hypoxia and normoxia^34,39–42^. A common observation is that levels of many EV proteins increase under hypoxic conditions, while very few proteins are down-regulated by hypoxia. However, the lists of proteins differentially expressed at hypoxia or normoxia vary widely between studies. There is practically no or little overlap in EV “hypoxia protein signatures” described for glioblastoma^40^, melanoma^41^, and combination of melanoma, squamous skin carcinoma and lung adenocarcinoma cells cultured in a hollow fiber system^39^.

Until now, no proteomics analysis of EVs produced by kidney cells cultured under hypoxia was published. The hypoxia-enriched proteins in Renca EVs were mostly associated with plasma membrane, as expected. There were several integrins induced by hypoxic treatment, which probably reflects changes in cell–cell adhesion relevant for cancer development^43^. The role of EV integrins in diverse pathophysiologic conditions in kidney such as tumor metastasis, neurological disorders, and immunology regulation has been recently reviewed^44^. Interestingly, we found similarities between EV proteins enriched under hypoxic conditions in our study, and the list of 61 proteins found only in cardiomyocytes-derived EVs cultured under hypoxia as compared to normoxia^34^. These common proteins include 4F2 cell-surface antigen heavy chain, Bone morphogenetic protein 1, and neutral amino acid transporter B(0).

The reason for major differences in results from various studies lies not only in the cell-type specific hypoxia responses, but apparently in diversity of EV purification methods used. The vast majority of studies describing hypoxia effects analyzed EVs isolated by either sequential ultracentrifugation or precipitation (see Table in^33^), that do co-purify wealth of contaminant proteins. Density gradient ultracentrifugation-based and size-exclusion chromatography-based isolation approaches, used in the current study, lead to isolation of better defined populations of EVs with less contaminants.

Novel methods for fast and reliable assessment of differences in EV composition, caused by external stimuli or resulting from different isolation approaches, are needed. We have recently reported a method for detecting different subsets of EVs with nuclear magnetic resonance (NMR)^45^. In the present study, we characterized EVs with Raman spectroscopy. While EVs could be detected also with spontaneous RS, SERS is the most common method applied to characterize EVs of different origins (see reviews^9,10^). Many variants of SERS, such as using gold nanoparticles, gold nanorods, silver nanocubes, and nano bowls, were successfully applied to describe composition of EVs. Another approach is the combination of SERS with EV capture by antibodies against exosomal EVs, such as CD63 and CD9, for example, in microfluidic Raman biochips^46^ and sandwich-based immunoassays^47,48^. These SERS studies demonstrated correlation between cancerous EVs and exosomal protein markers^49^. It was recently shown that TG-SERS with gold nanoparticles could distinguish EVs produced by red blood cells from those generated by platelets^26^.

In the present study, we developed new TG-RS and TG-SERS methods that detected differences in composition between EVs produced under hypoxic and normoxic conditions in vitro. For the separation between normoxic and hypoxic condition by TG-RS with and without SERS, two protein associated areas appeared to be interesting, the first one amide IV from 750 to 1000 cm^–1^, dominated by proteins and nucleic acids, and the second one, amide III from 1300 to 1600 cm^–1^ dominated by lipids, proteins, nucleotides and other DNA/RNA macromolecules. The later area in the amide III (assigned e.g. to lipids or cholesterol), around 1405, 1440 and 1455 cm^–1^ reveal in our TG-RS and TG-SERS measurements a noticeable heterogeneity across normoxic and hypoxic conditions and the different isolation methods. Among these bands, there are nucleic acid base and CH/CH_2_ deformations.

RS gives a classifying “fingerprint” of an EV sample, indicating changes in proteins, lipids, and nucleic acids, while Mass spectroscopy provides much more detailed analysis of sample molecular composition. While RS does not offer as extensive information about EV samples as quantitative proteomics, it has clear advantages towards clinical diagnostics, due to its relative rapidness and lower workload^51^. Though it is rather challenging to make direct comparison between results obtained by using RS and proteomics studies, certain spectral peaks noted in RS may indicate characteristic features of protein composition. For instance, hemoproteins and iron-containing proteins in general could be detected by RS (Table 1;^52,53^). Heme-containing proteins such as catalase and cytochrome C were identified by proteomics analysis of EV samples in our study, and iron storage protein ferritin is one of the most abundant proteins found in RCC EV samples by proteomics. There are similarities in the RS spectra observed in CD9-positive EV subpopulation compared to total EVs, as pointed out by Carney and co-workers^54^, and in our study. Importantly, increase in CD9-positive EVs upon hypoxia treatment was found both by Western blotting and proteomics analysis.

It is known that EV composition and biological activity strongly depend on the purification method used, which limits ability to compare results from different studies^55^. Indeed, we found that different purification methods seem to have even stronger influence on the EV RS results as compared to hypoxia treatment, indicating that the Raman technology is a useful way to assess the EV purification methods and their applicability. It is known that gradient-based purification as compared to, for example, size-exclusion chromatography, usually result in a more homogenous EV population with higher proportion of EVs expressing exosomal markers and less contaminants, which is true also for our study (Fig. 2; Supplementary Figure S4). On the other hand, EV yields after gradient purification are often not sufficient for many applications, especially proteomics and transcriptomics that require a lot of starting material for quantitative analysis. In our study, gradient-based isolation resulted in about 8–9 times lower EV protein yields from the same starting number of cultured cells. CW-RS was previously shown to be a quick and reliable method to assess purity of EVs isolated by conventional ultracentrifugation (UC) versus size exclusion chromatography (SEC)^55^. Now we demonstrate that TG-RS gives reliable analysis of gradient EV samples and could be used for its comparison with EVs purified by SEC.

We conclude that Time-gated RS, with and without SERS, provides relatively fast, label free method to assay stimuli induced differences in EV molecular composition as well as differences resulting from selected EV purification methods, while proteomics serves as a tool to depict the changes of individual proteins amounts in the same samples. Both pre-clinical and clinical studies will be needed to obtain evidence whether proposed methods may be useful to characterize EVs isolated from human biofluids.

## Conclusions

EVs released by RCC cells of mouse (Renca) and human (786-O) origin cultured under hypoxia and normoxia were characterized by combination of TG-RS, TG-SERS, proteomics, Western blot analysis, NTA, and electron microscopy. A number of proteins, especially those involved in cell adhesion, were overrepresented in EVs produced by Renca cells cultured under hypoxia. We show that TG-RS provides a powerful tool to overcome the problem of background fluorescence, that is typically masking the Raman signal of most biological specimen, and that TG-RS signal can still be further improved with SERS. EVs isolated by using two different isolation methods had strikingly different RS spectra. Therefore, the combination of TG-RS and TG-SERS has the potential to provide insights in qualitative changes of EV content in response to stimuli such as hypoxia and also in assaying purity of EV isolations.

## Supplementary Information

Supplementary Information.

Supplementary Table S2.

Supplementary Table S3.

## Data Availability

The mass spectrometry proteomics data have been deposited to the ProteomeXchange Consortium via the PRIDE^56^ partner repository with the dataset identifier PXD023546 and https://doi.org/10.6019/PXD023546.

## References

[1] Hsieh JJ (2017). Renal cell carcinoma. Nat. Rev. Dis. Prim..

[2] Calvo E, Porta C, Grünwald V, Escudier B (2019). The current and evolving landscape of first-line treatments for advanced renal cell carcinoma. Oncologist.

[3] Van Niel G, D’Angelo G, Raposo G (2018). Shedding light on the cell biology of extracellular vesicles. Nat. Rev. Mol. Cell Biol..

[4] Kalluri R, LeBleu VS (2020). The biology, function, and biomedical applications of exosomes. Science..

[5] Kumar A, Deep G (2020). Exosomes in hypoxia-induced remodeling of the tumor microenvironment. Cancer Lett..

[6] Semenza GL (2019). Pharmacologic targeting of hypoxia-inducible factors. Annu. Rev. Pharmacol. Toxicol..

[7] Banks RE (2006). Genetic and epigenetic analysis of von Hippel–Lindau (VHL) gene alterations and relationship with clinical variables in sporadic renal cancer. Cancer Res..

[8] Zhang Y, Mi X, Tan X, Xiang R (2019). Recent progress on liquid biopsy analysis using surface-enhanced Raman spectroscopy. Theranostics.

[9] Merdalimova A (2019). Identification and analysis of exosomes by surface-enhanced Raman spectroscopy. Appl. Sci..

[10] Rojalin T, Phong B, Koster H, Carney RP (2019). Nanoplasmonic approaches for sensitive detection and molecular characterization of extracellular vesicles. Front. Chem..

[11] Kögler M (2018). Bare laser-synthesized Au-based nanoparticles as nondisturbing surface-enhanced Raman scattering probes for bacteria identification. J. Biophotonics.

[12] Procházka, M. *Surface Enhanced Raman Spectroscopy*. *Biological and Medical Physics, Biomedical Engineering* (Springer, Berlin, 2016). 10.1007/978-3-319-23992-7.

[13] Panagopoulou MS, Wark AW, Birch DJS, Gregory CD (2020). Phenotypic analysis of extracellular vesicles: A review on the applications of fluorescence. J. Extracell. Vesicles.

[14] Kögler M (2018). Comparison of time-gated surface-enhanced Raman spectroscopy (TG-SERS) and classical SERS based monitoring of *Escherichia coli* cultivation samples. Biotechnol. Prog..

[15] Kögler, M., Itkonen, J., Viitala, T. & Casteleijn, M. G. Assessment of recombinant protein production in *E. coli* with time-gated surface enhanced Raman spectroscopy (TG-SERS). *Sci. Rep.* **10**, 2472 (2020). 10.1038/s41598-020-59091-3 PMC7015922 32051493

[16] Lipiäinen T (2018). Time-gated Raman spectroscopy for quantitative determination of solid-state forms of fluorescent pharmaceuticals. Anal. Chem..

[17] Rojalin T (2016). Fluorescence-suppressed time-resolved Raman spectroscopy of pharmaceuticals using complementary metal-oxide semiconductor (CMOS) single-photon avalanche diode (SPAD) detector. Anal. Bioanal. Chem..

[18] Kögler, M. & Heilala, B. Time-gated Raman spectroscopy—A review. *Meas. Sci. Technol.* **32**, 012002 (2020).

[19] Théry C (2018). Minimal information for studies of extracellular vesicles 2018 (MISEV2018): A position statement of the International Society for Extracellular Vesicles and update of the MISEV2014 guidelines. J. Extracell. Vesicles.

[20] Van Deun J (2017). EV-TRACK: Transparent reporting and centralizing knowledge in extracellular vesicle research. Nat. Methods.

[21] Supek F, Bošnjak M, Škunca N, Šmuc T (2011). Revigo summarizes and visualizes long lists of gene ontology terms. PLoS One.

[22] Andreu Z, Yáñez-Mó M (2014). Tetraspanins in extracellular vesicle formation and function. Front. Immunol..

[23] Arroyo JD (2011). Argonaute2 complexes carry a population of circulating microRNAs independent of vesicles in human plasma. Proc. Natl. Acad. Sci. USA.

[24] David, C. Raman Spectroscopy for proteins. *Horiba Scientific Webminar*17–53 (2012).

[25] Rygula A (2013). Raman spectroscopy of proteins: A review. J Raman Spectrosc..

[26] Koponen A (2020). Label-free characterization and real-time monitoring of cell uptake of extracellular vesicles. Biosens. Bioelectron..

[27] Szekeres GP, Kneipp J (2019). SERS probing of proteins in gold nanoparticle agglomerates. Front. Chem..

[28] Notingher I (2007). Raman spectroscopy cell-based biosensors. Sensors.

[29] De Gelder J, De Gussem K, Vandenabeele P, Moens L (2007). Reference database of Raman spectra of biological molecules. J Raman Spectrosc..

[30] Jing X (2019). Role of hypoxia in cancer therapy by regulating the tumor microenvironment. Mol. Cancer.

[31] Kumar A, Deep G (2020). Hypoxia in tumor microenvironment regulates exosome biogenesis: Molecular mechanisms and translational opportunities. Cancer Lett..

[32] Shao C (2018). Role of hypoxia-induced exosomes in tumor biology. Mol Cancer.

[33] Zonneveld MI, Keulers TGH, Rouschop KMA (2019). Extracellular vesicles as transmitters of hypoxia tolerance in solid cancers. Cancers.

[34] Ontoria-Oviedo I (2018). Extracellular vesicles secreted by hypoxic ac10 cardiomyocytes modulate fibroblast cell motility. Front Cardiovasc Med..

[35] Zhang W (2017). HIF-1-mediated production of exosomes during hypoxia is protective in renal tubular cells. Am J Physiol Ren Physiol..

[36] Maxwell PH (1999). The tumour suppressor protein VHL targets hypoxia-inducible factors for oxygen-dependent proteolysis. Nature.

[37] Patton MC, Zubair H, Khan MA, Singh S, Singh AP (2020). Hypoxia alters the release and size distribution of extracellular vesicles in pancreatic cancer cells to support their adaptive survival. J Cell Biochem..

[38] Gardiner C, Ferreira YJ, Dragovic RA, Redman CWG, Sargent IL (2013). Extracellular vesicle sizing and enumeration by nanoparticle tracking analysis. J Extracell Vesicles.

[39] Park JE (2019). Hypoxia-induced tumor exosomes promote M2-like macrophage polarization of infiltrating myeloid cells and microRNA-mediated metabolic shift. Oncogene.

[40] Indira Chandran V (2019). Global extracellular vesicle proteomic signature defines U87-MG glioma cell hypoxic status with potential implications for non-invasive diagnostics. J Neurooncol..

[41] Walbrecq G (2020). Hypoxia-induced adaptations of mirnomes and proteomes in melanoma cells and their secreted extracellular vesicles. Cancers.

[42] Horie K (2017). Exosomes expressing carbonic anhydrase 9 promote angiogenesis. Biochem Biophys Res Commun..

[43] Ata R, Antonescu CN (2017). Integrins and cell metabolism: An intimate relationship impacting cancer. Int J Mol Sci..

[44] Chen, H. *et al. Integrin, Exosome and Kidney Disease*. (Frontiers Media S.A., 2021). 10.3389/fphys.2020.627800. 10.3389/fphys.2020.627800 PMC7868550 33569013

[45] Ullah MS (2021). Identification of extracellular nanoparticle subsets by nuclear magnetic resonance. Chem Sci..

[46] Wang Y (2020). Microfluidic Raman biochip detection of exosomes: A promising tool for prostate cancer diagnosis. Lab Chip.

[47] Li TD (2018). An ultrasensitive polydopamine bi-functionalized SERS immunoassay for exosome-based diagnosis and classification of pancreatic cancer. Chem Sci..

[48] Weng Z (2018). Screening and multiple detection of cancer exosomes using an SERS-based method. Nanoscale.

[49] Shin H, Jeong H, Park J, Hong S, Choi Y (2018). Correlation between cancerous exosomes and protein markers based on surface-enhanced Raman spectroscopy (SERS) and principal component analysis (PCA). ACS Sensors.

[50] Cui L, Butler HJ, Martin-Hirsch PL, Martin FL (2016). Aluminium foil as a potential substrate for ATR-FTIR, transflection FTIR or Raman spectrochemical analysis of biological specimens. Anal Methods.

[51] Aitekenov S, Gaipov A, Bukasov R (2021). Review: Detection and quantification of proteins in human urine. Talanta.

[52] Kitahama Y, Ozaki Y (2016). Surface-enhanced resonance Raman scattering of hemoproteins and those in complicated biological systems. Analyst.

[53] Ruvalcaba-López JM (2019). Qualitative evaluation of ferritin in serum samples by Raman spectroscopy and principal component analysis. Lasers Med Sci..

[54] Carney RP (2017). Multispectral optical tweezers for biochemical fingerprinting of CD9-positive exosome subpopulations. Anal Chem..

[55] Gualerzi A (2019). Raman spectroscopy as a quick tool to assess purity of extracellular vesicle preparations and predict their functionality. J Extracell Vesicles.

[56] Perez-Riverol Y (2019). The PRIDE database and related tools and resources in 2019: Improving support for quantification data. Nucleic Acids Res..

[57] Hornemann A, Drescher D, Flemig S, Kneipp J (2013). Intracellular SERS hybrid probes using BSA-reporter conjugates optical nanosensing in cells. Anal Bioanal Chem..

[58] Stremersch S (2016). Identification of individual exosome-like vesicles by surface enhanced raman spectroscopy. Small.

[59] Gualerzi A (2017). Raman spectroscopy uncovers biochemical tissue-related features of extracellular vesicles from mesenchymal stromal cells. Sci Rep..

[60] Radziuk D, Moehwald H (2014). Highly effective hot spots for SERS signatures of live fibroblasts. Nanoscale.

[61] Lednev, I. K. *Application of Raman Spectroscopy for an Easy-to-use, on-Field, Rapid, Nondestructive, Confirmatory Identification of Body Fluids*. *Report US Department of Justice* (2012).

