# Supplementary material for: Time-gated Raman spectroscopy and proteomics analyses of hypoxic and normoxic renal carcinoma extracellular vesicles
Source: Sci Rep. 2021 Oct 1;11:19594. doi: 10.1038/s41598-021-99004-6 (PMC8486794; doi:10.1038/s41598-021-99004-6)
Supplement: Supplementary file 1 — Supplementary Information. [file 41598_2021_99004_MOESM1_ESM.pdf]

Time-gated Raman spectroscopy and proteomics analyses of hypoxic and normoxic renal carcinoma extracellular vesicles

Anatoliy Samoylenko[1]*, Martin Kögler[2], Artem Zhyvolozhnyi[1], Olha Makieieva[1], Genevieve Bart[1], Sampson S. Andoh[3], Matthieu Roussey[3], Seppo J. Vainio[1], Jussi Hiltunen[2].
Corresponding Author*

---

[1] Faculty of Biochemistry and Molecular Medicine, Disease Networks Research Unit, Laboratory of Developmental Biology, Kvantum Institute, Infotech Oulu, 90014 Oulu University of Oulu, Oulu, Finland
[2] VTT Technical Research Centre of Finland, 90570 Oulu, Finland
[3] Institute of Photonics, University of Eastern Finland, 80101 Joensuu, Finland

*Correspondence
Anatoliy Samoylenko, University of Oulu, 90014, Oulu, Finland
tel. +358442423312
Email: anatoliy.samoylenko@oulu.fi

Supporting Information

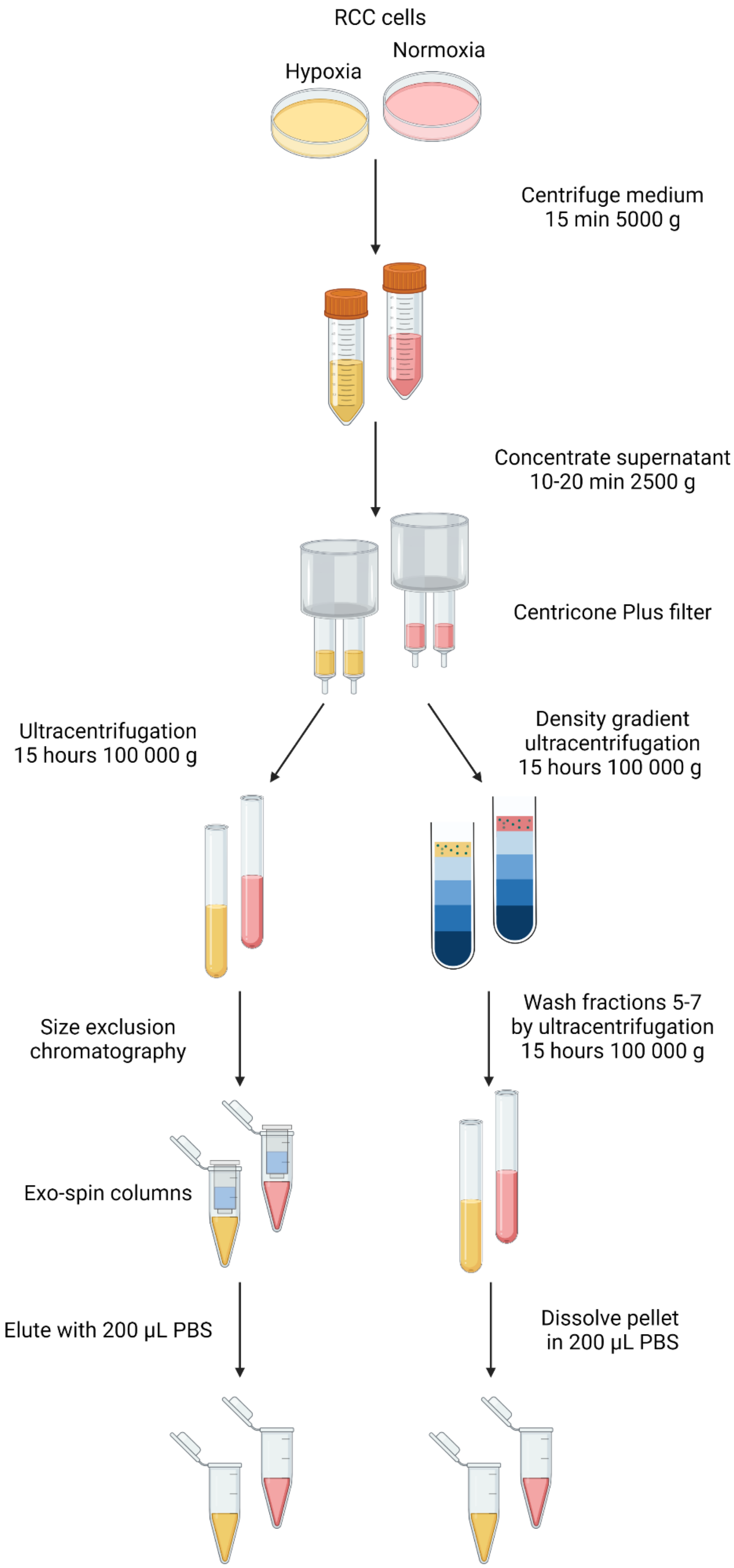

Suppl. Figure 1. Workflow of EV isolations from cell culture media. Image created with BioRender.com.

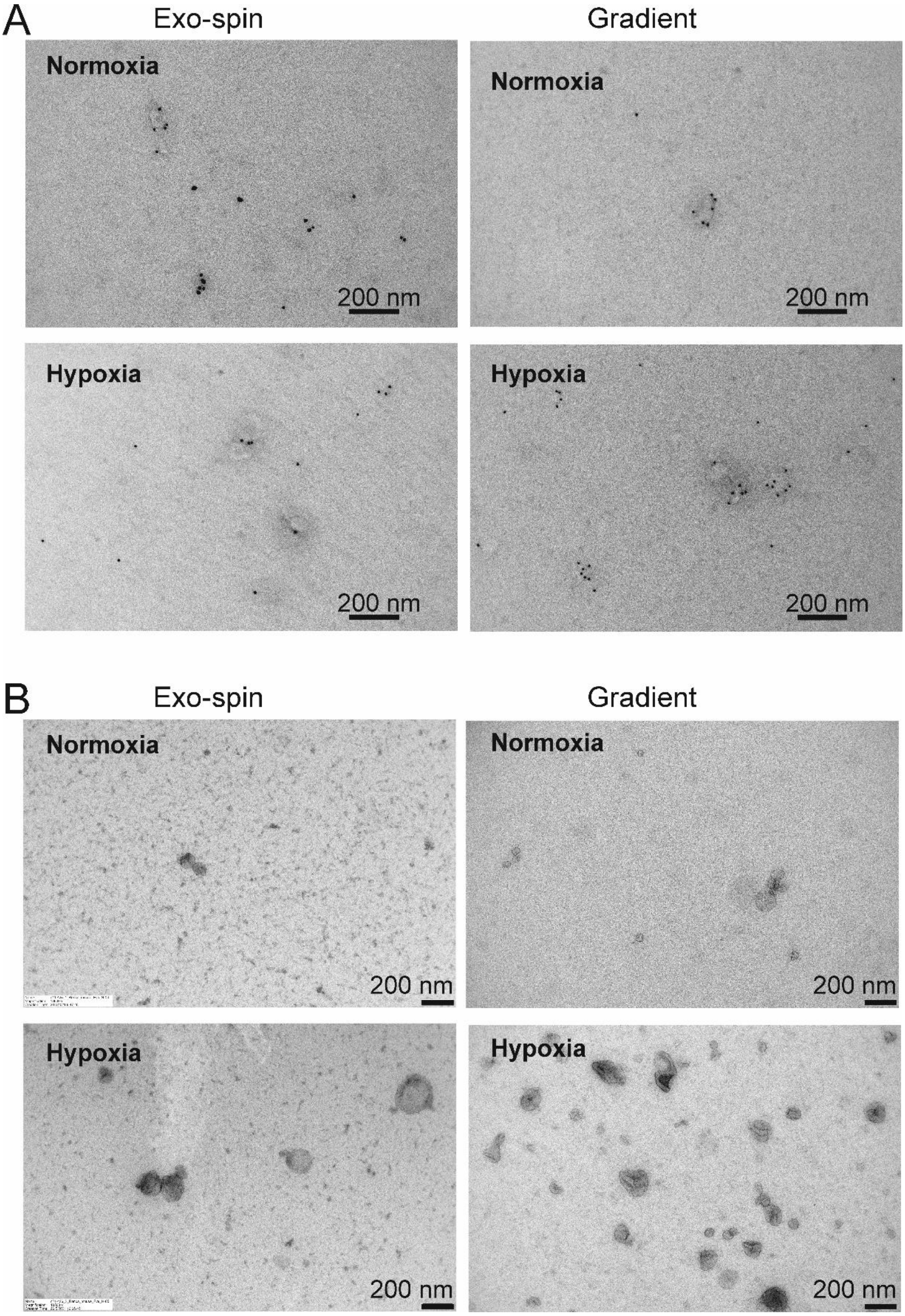

Suppl. Figure 2. Immuno-TEM with anti-CD63 antibody (A) and negative staining TEM (B) of Renca EV samples.

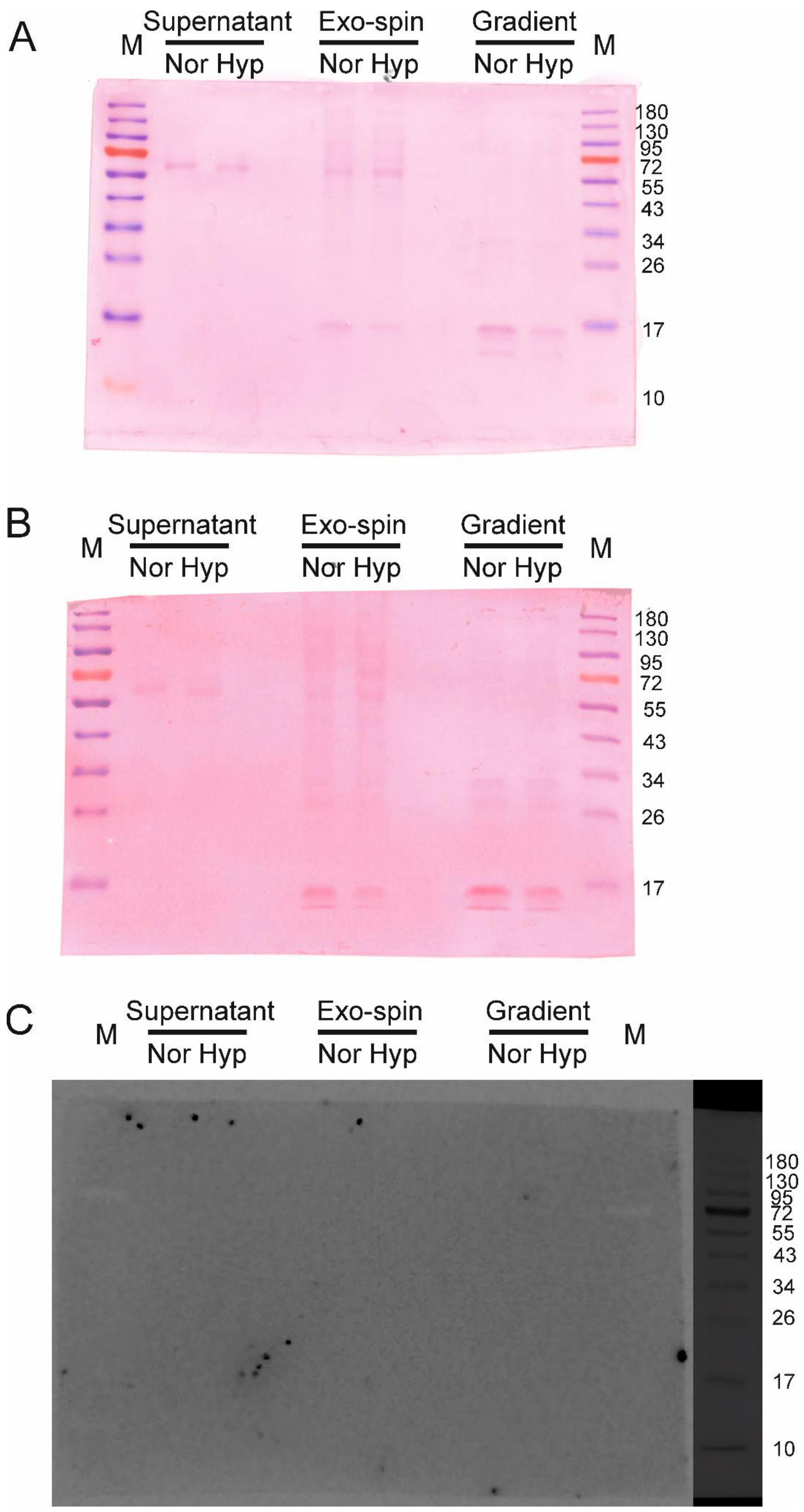
A
M
Supernatant
Nor Hyp
Exo-spin
Nor Hyp
Gradient
Nor Hyp
M
180
130
95
72
55
43
34
26
17
10
B
M
Supernatant
Nor Hyp
Exo-spin
Nor Hyp
Gradient
Nor Hyp
M
180
130
95
72
55
43
34
26
17
C
M
Supernatant
Nor Hyp
Exo-spin
Nor Hyp
Gradient
Nor Hyp
M
180
130
95
72
55
43
34
26
17
10

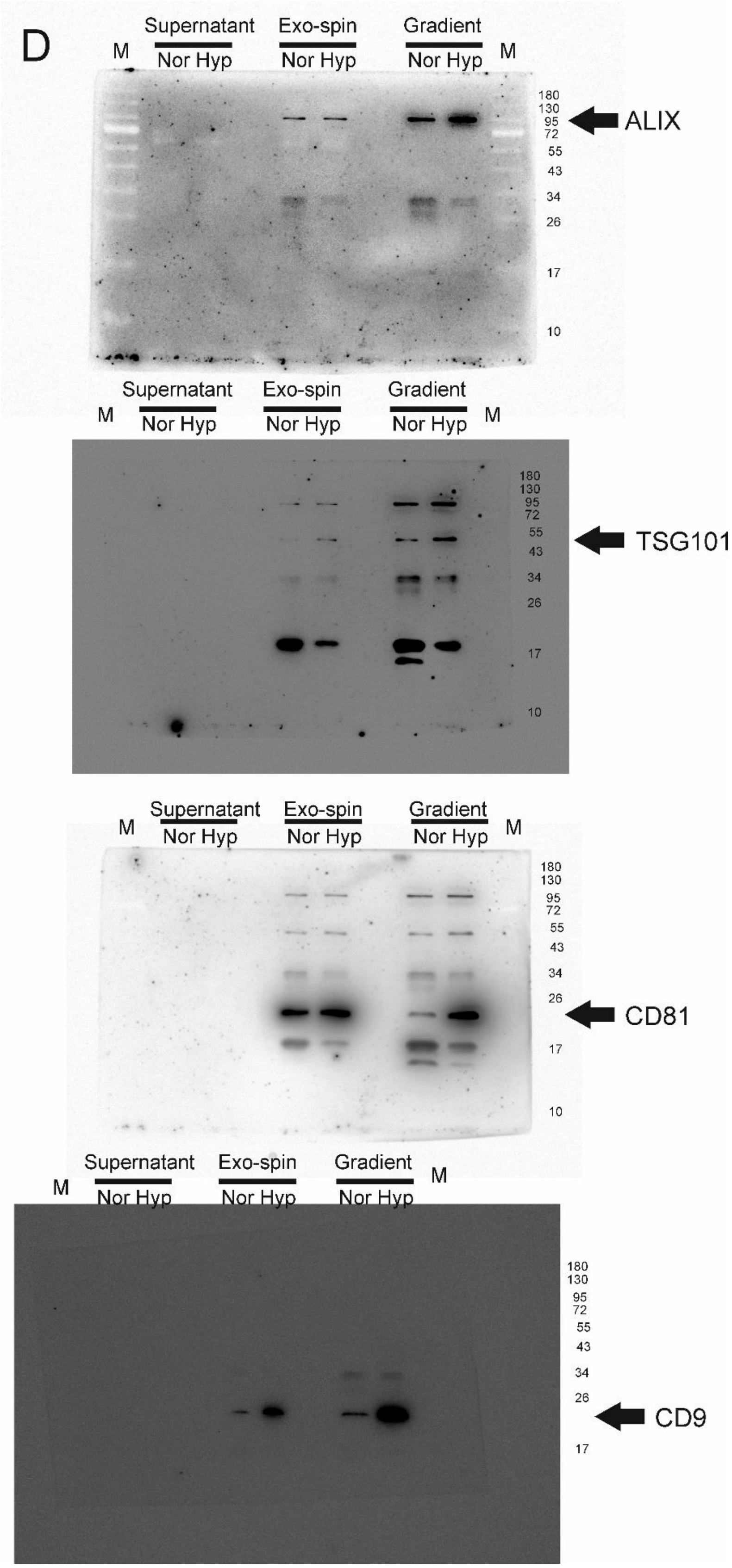

Suppl. Figure 3. Ponceau S (Sigma-Aldrich) staining of membranes used for Western blotting with (A): Alix, TSG101, and (B): CD81, CD9 (corresponding blots are shown in Figure 2C). (C) Western blot with antibody against Argonaute2 (Ago2). (D) Images of uncropped blots used for Figure 2C. M – marker (PageRuler Prestained Protein Ladder, Thermo Fisher, 26616).

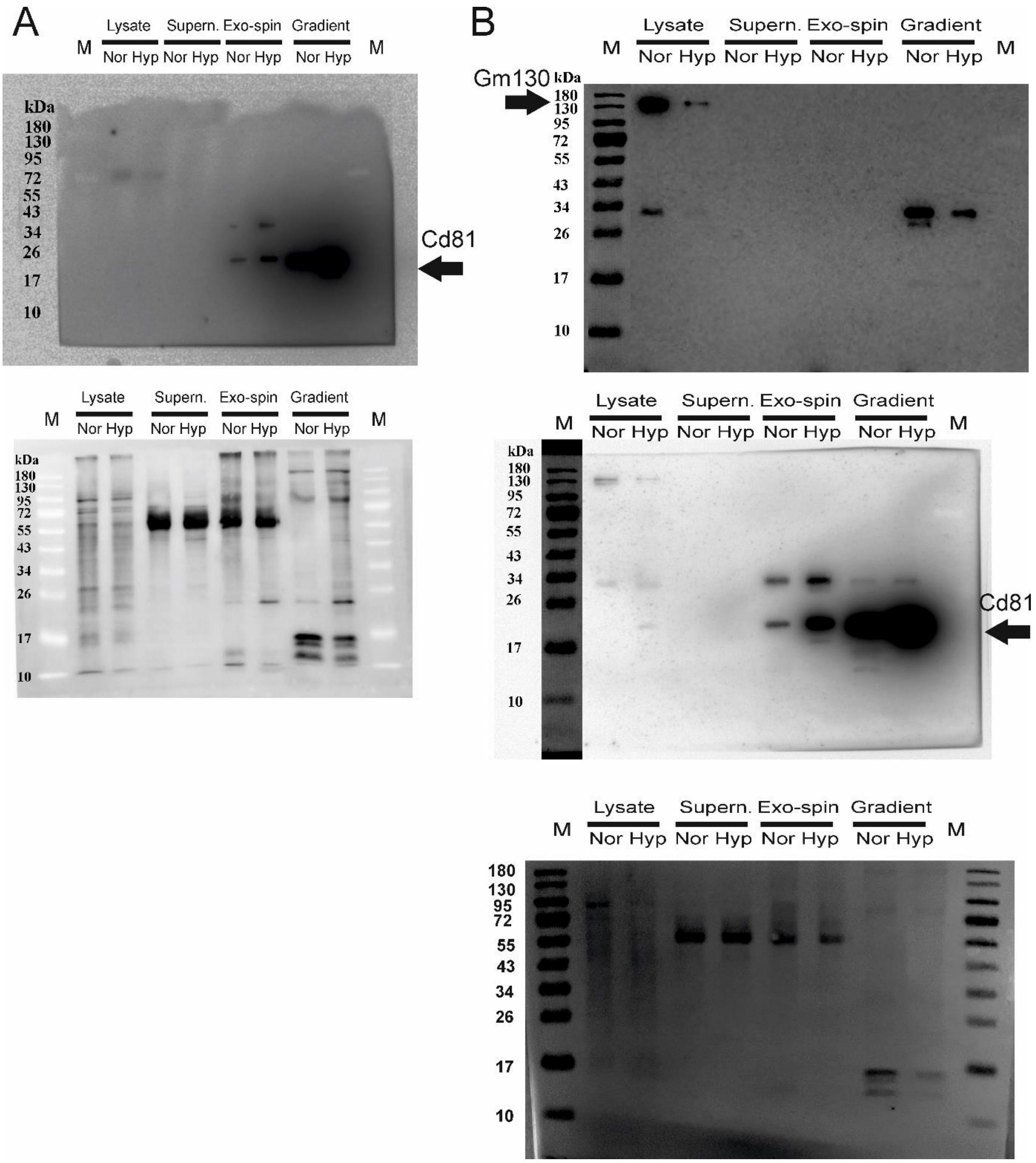

Suppl. Figure 4. (A) Western blot with anti-CD81 antibody (top) and total protein stain of the same membrane (Azure Biosystems). (B) Western blot with anti-GM130 antibody (top), anti-CD81 antibody (middle) and Ponceau S (Sigma-Aldrich) protein staining of the same membrane. M – marker (PageRuler Prestained Protein Ladder, Thermo Fisher, 26616).

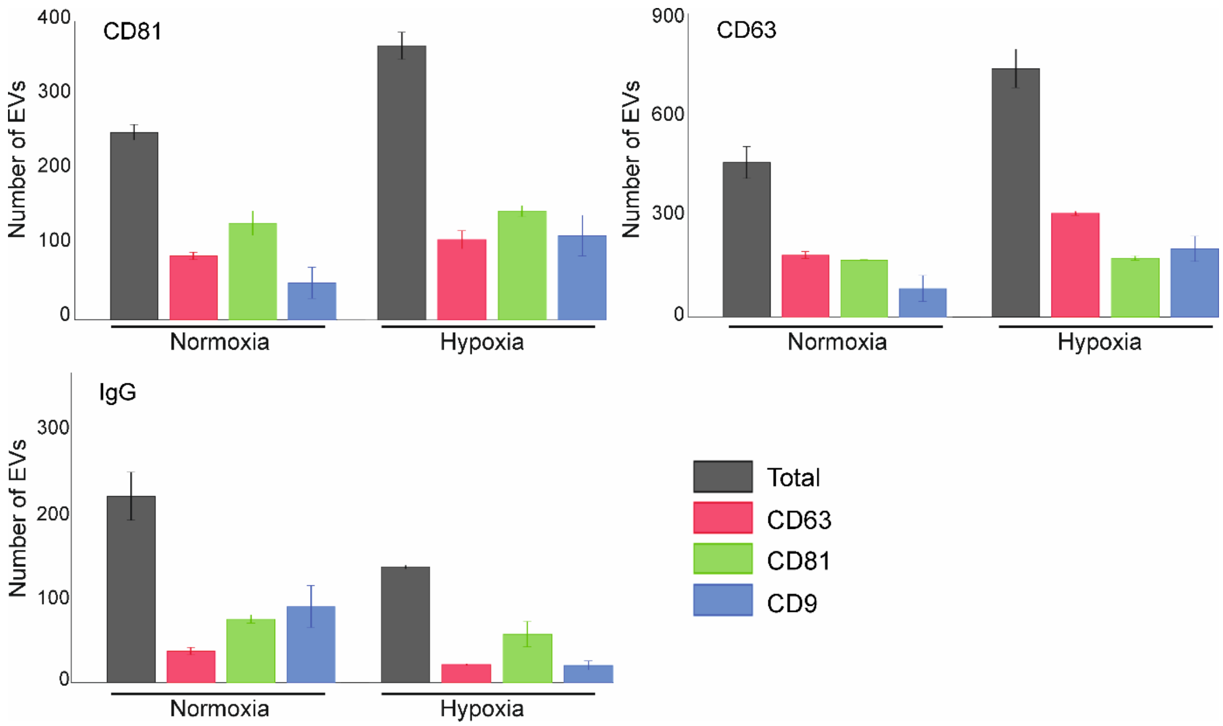

Suppl. Figure 5. Characterization of EVs produced by 786-O cells cultured under normoxia or hypoxia in cell culture supernatants by ExoView. Names of the capture chips are shown in the upper left corners, total number of detected EVs in a sample (1 µg total protein) are shown by grey bars, number of EVs expressing CD63, CD81, and CD9 by red, green, and blue bars, correspondingly.

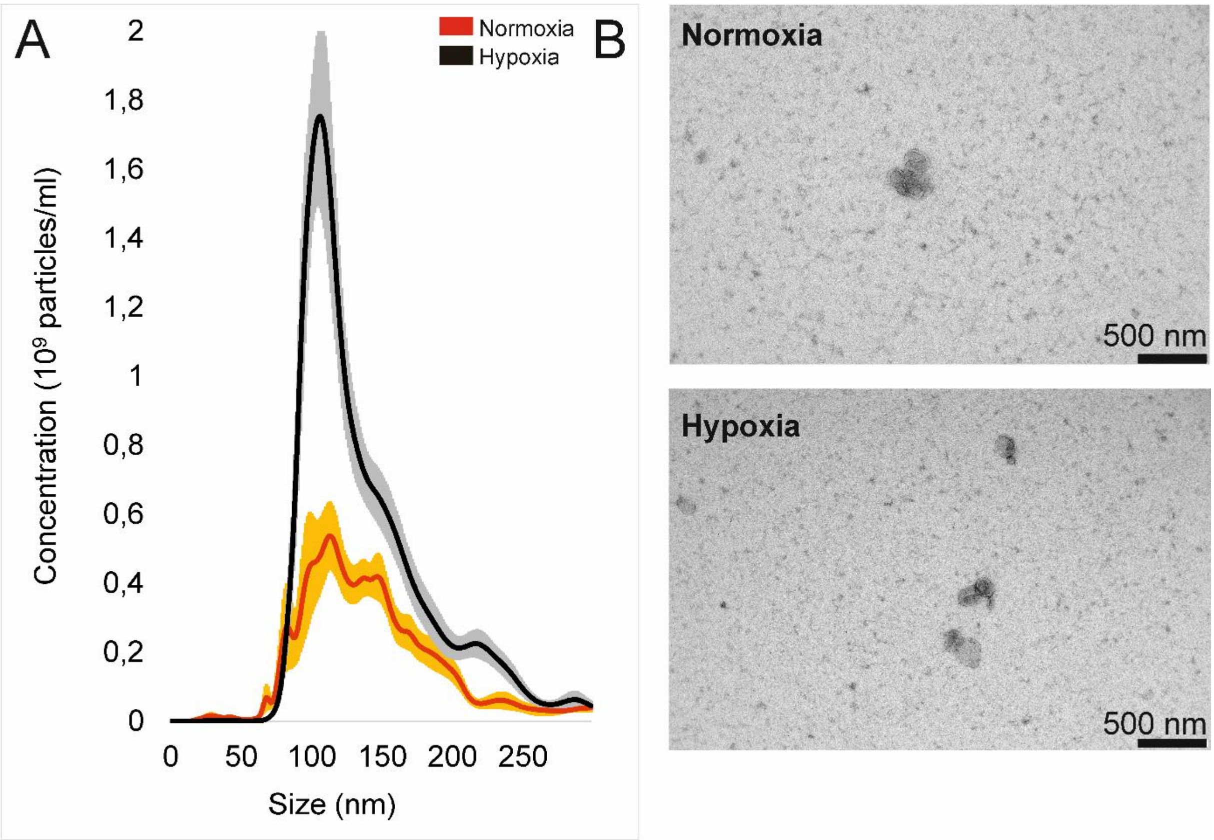

Suppl. Figure 6. Analysis of EVs produced by 786-O RCC cells under hypoxia and normoxia by NTA (A) and TEM (B).

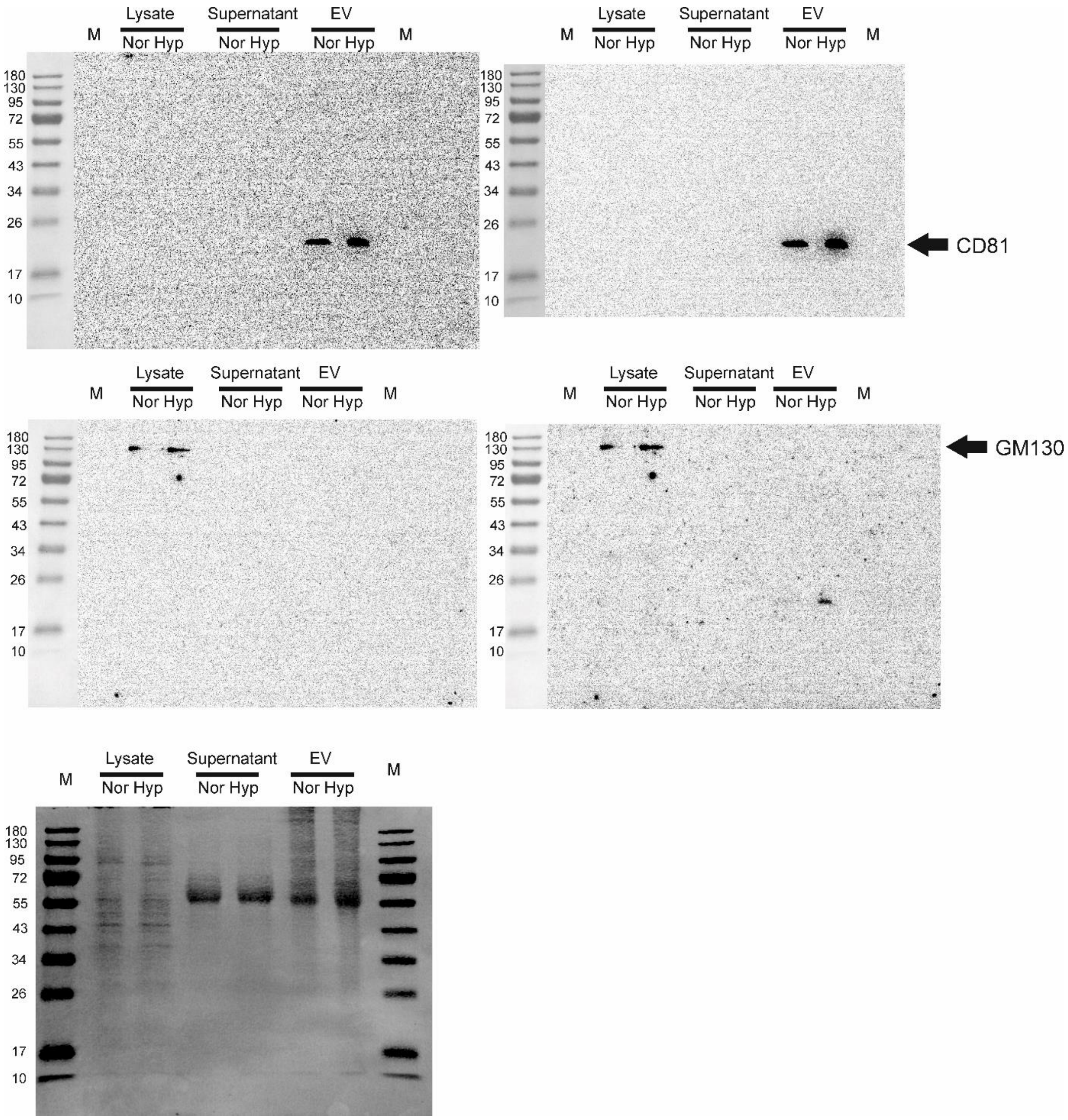

Suppl. Figure 7. Western blots with EVs from human 786-O cells using anti-CD81 antibody (2 min and 5 min exposure) and anti-GM130 antibody (10 min and 30 min exposure). Lower image shows Ponceau S (Sigma-Aldrich) staining of membrane used for Western blotting.

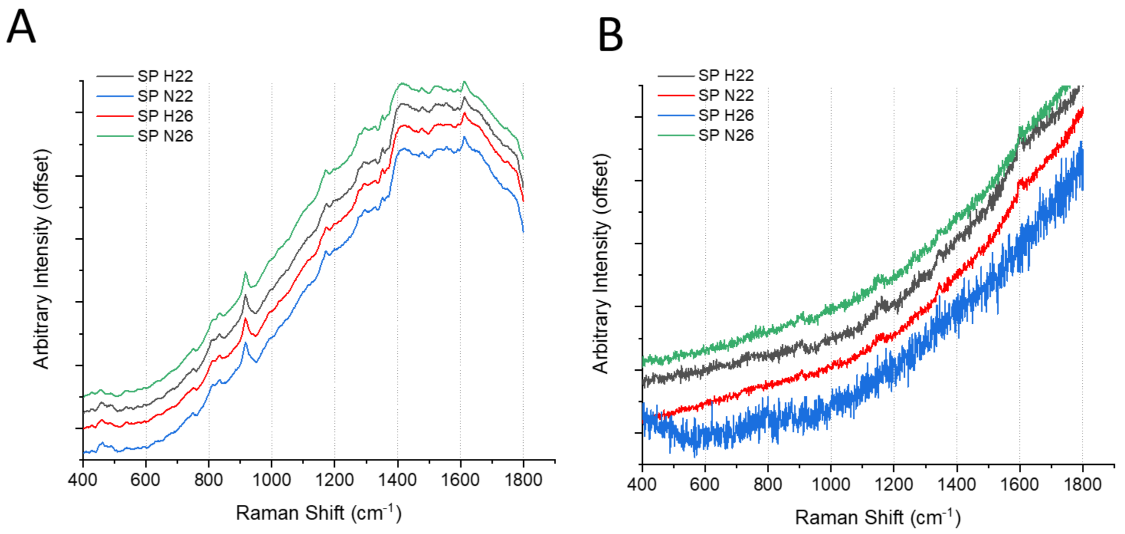

Suppl. Figure 8. Measurements of EV-depleted supernatants (SP) with very high fluorescence background. (A) TG-RS with some noticeable peaks and (B) CW-Raman without any distinguishable signals.

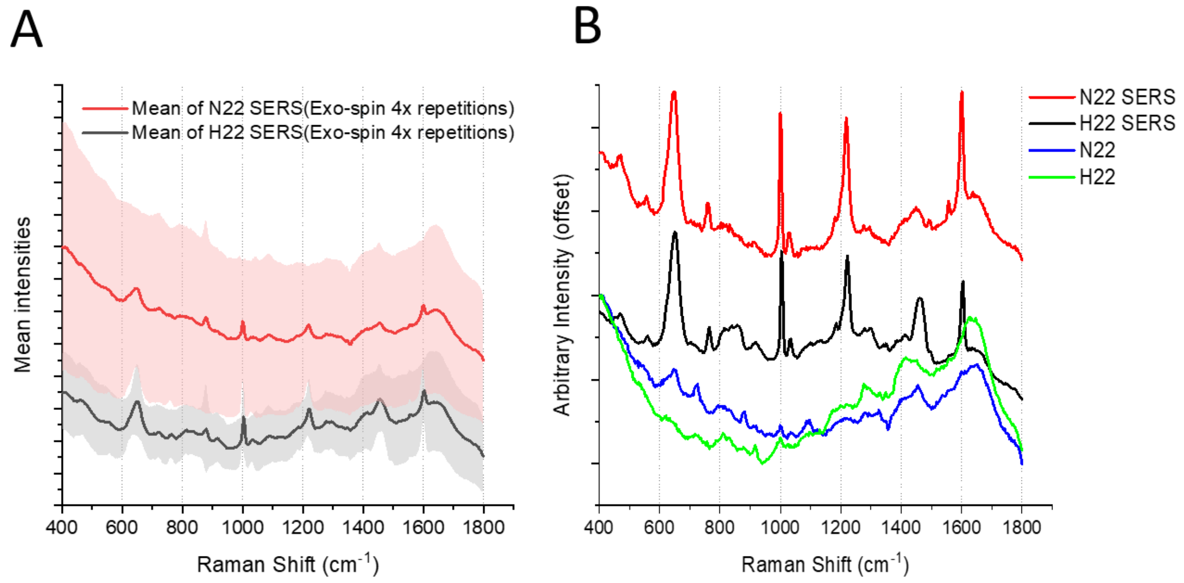

Suppl. Figure 9. (A) Variations in TG-SERS measurements of normoxic (N, in red) and hypoxic (H, in black) EV samples. (B) Comparison of TG-SERS (N, normoxia, in black; H, hypoxia, in red) and TG-RS (N, normoxia, in blue; H, hypoxia, in green) spectra of samples N22 and H22, isolated by using Exo-spin.

Suppl. Table 1. Proteins identified in EV samples by mass spectrometry (corresponds to the data available via ProteomeXchange with identifier PXD023546).

Suppl. Table 2. Proteins identified in EV-depleted cell culture media samples by mass spectrometry (corresponds to the data available via ProteomeXchange with identifier PXD023546).

PRIDE database
Submission details:
Project Name: Renal Cell Carcinoma (Renca) Extracellular Vesicles under Hypoxia and Normoxia
Project accession: PXD023546
Project DOI: 10.6019/PXD023546

Suppl. Table 3. GO analysis for "Biological processes" (all EV proteins)

Term ID	Description	Number	Fold enrichment	p value
GO:0006188	IMP biosynthetic process	7	16,10	3.81E-02
GO:0046040	IMP metabolic process	9	15,06	2.25E-03
GO:1904851	positive regulation of establishment of protein localization to telomere	8	14,72	1.20E-02
GO:0010499	proteasomal ubiquitin-independent protein catabolic process	17	14,22	1.79E-08
GO:0070203	regulation of establishment of protein localization to telomere	8	13,38	1.99E-02
GO:0009168	purine ribonucleoside monophosphate biosynthetic process	14	12,88	4.11E-06
GO:0009127	purine nucleoside monophosphate biosynthetic process	14	12,88	4.11E-06
GO:0070202	regulation of establishment of protein localization to chromosome	8	12,27	3.16E-02
GO:0009156	ribonucleoside monophosphate biosynthetic process	16	12,27	3.93E-07
GO:0019081	viral translation	9	11,83	9.49E-03
GO:0042730	fibrinolysis	9	11,83	9.49E-03
GO:1904816	positive regulation of protein localization to chromosome, telomeric region	8	11,32	4.87E-02
GO:0051131	chaperone-mediated protein complex assembly	10	10,82	4.32E-03
GO:0009124	nucleoside monophosphate biosynthetic process	17	10,79	4.35E-07
GO:1901673	regulation of mitotic spindle assembly	10	10,22	6.41E-03
GO:0009167	purine ribonucleoside monophosphate metabolic process	16	10,15	3.17E-06
GO:0009161	ribonucleoside monophosphate metabolic process	18	10,04	2.89E-07
GO:0009126	purine nucleoside monophosphate metabolic process	16	9,81	4.63E-06
GO:0098761	cellular response to interleukin-7	9	9,74	3.15E-02
GO:0098760	response to interleukin-7	9	9,74	3.15E-02
GO:0006541	glutamine metabolic process	10	9,68	9.34E-03
GO:0002181	cytoplasmic translation	34	0,42	1.41E-15
GO:0006418	tRNA aminoacylation for protein translation	19	0,38	5.18E-07
GO:0009123	nucleoside monophosphate metabolic process	19	8,53	7.22E-07
GO:0006413	translational initiation	25	8,36	1.06E-09
GO:0043039	tRNA aminoacylation	19	8,13	1.37E-06
GO:0090169	regulation of spindle assembly	11	8,10	1.07E-02
GO:1901068	guanosine-containing compound metabolic process	13	7,97	1.31E-03
GO:0043038	amino acid activation	19	7,95	1.87E-06
GO:0006457	protein folding	56	6,87	1.60E-21
GO:0006412	translation	112	6,76	1.55E-45
GO:0061077	chaperone-mediated protein folding	19	6,72	1.78E-05
GO:0006458	'de novo' protein folding	13	6,64	7.06E-03
GO:0051084	'de novo' posttranslational protein folding	13	6,64	7.06E-03
GO:0046128	purine ribonucleoside metabolic process	18	6,62	5.72E-05
GO:0009116	nucleoside metabolic process	27	6,62	9.79E-09
GO:0032509	endosome transport via multivesicular body sorting pathway	12	6,49	2.28E-02
GO:1901607	alpha-amino acid biosynthetic process	19	6,47	2.96E-05
GO:0043043	peptide biosynthetic process	114	6,47	6.75E-45
GO:0042255	ribosome assembly	23	6,41	7.83E-07

Term ID	Description	Number	Fold enrichment	p value
GO:0060236	regulation of mitotic spindle organization	12	6,31	2.92E-02
GO:0009112	nucleobase metabolic process	12	6,31	2.92E-02
GO:1904358	positive regulation of telomere maintenance via telomere lengthening	12	6,31	2.92E-02
GO:0009119	ribonucleoside metabolic process	19	6,24	4.84E-05
GO:0019058	viral life cycle	26	6,21	8.37E-08
GO:1901657	glycosyl compound metabolic process	33	6,20	1.46E-10
GO:0042278	purine nucleoside metabolic process	18	6,13	1.53E-04
GO:0008652	cellular amino acid biosynthetic process	19	6,03	7.77E-05
GO:0006518	peptide metabolic process	144	5,94	5.59E-54
GO:1900047	negative regulation of hemostasis	14	5,60	1.45E-02
GO:0043604	amide biosynthetic process	128	5,54	1.04E-44
GO:0050819	negative regulation of coagulation	14	5,48	1.80E-02
GO:0016032	viral process	40	5,41	1.10E-11
GO:0050818	regulation of coagulation	20	5,33	1.78E-04
GO:1900046	regulation of hemostasis	19	5,30	4.39E-04
GO:0042273	ribosomal large subunit biogenesis	21	5,22	1.08E-04
GO:1900024	regulation of substrate adhesion-dependent cell spreading	15	5,11	1.63E-02
GO:0030193	regulation of blood coagulation	18	5,10	1.61E-03
GO:0016052	carbohydrate catabolic process	24	5,08	1.60E-05
GO:0071826	ribonucleoprotein complex subunit organization	49	5,07	6.61E-14
GO:0060236	regulation of mitotic spindle organization	12	6,31	2.92E-02
GO:0009112	nucleobase metabolic process	12	6,31	2.92E-02
GO:1904358	positive regulation of telomere maintenance via telomere lengthening	12	6,31	2.92E-02
GO:0009119	ribonucleoside metabolic process	19	6,24	4.84E-05
GO:0019058	viral life cycle	26	6,21	8.37E-08
GO:1901657	glycosyl compound metabolic process	33	6,20	1.46E-10
GO:0042278	purine nucleoside metabolic process	18	6,13	1.53E-04
GO:0008652	cellular amino acid biosynthetic process	19	6,03	7.77E-05
GO:0006518	peptide metabolic process	144	5,94	5.59E-54
GO:1900047	negative regulation of hemostasis	14	5,60	1.45E-02
GO:0043604	amide biosynthetic process	128	5,54	1.04E-44
GO:0050819	negative regulation of coagulation	14	5,48	1.80E-02
GO:0016032	viral process	40	5,41	1.10E-11
GO:0050818	regulation of coagulation	20	5,33	1.78E-04
GO:1900046	regulation of hemostasis	19	5,30	4.39E-04
GO:0042273	ribosomal large subunit biogenesis	21	5,22	1.08E-04
GO:1900024	regulation of substrate adhesion-dependent cell spreading	15	5,11	1.63E-02
GO:0030193	regulation of blood coagulation	18	5,10	1.61E-03
GO:0016052	carbohydrate catabolic process	24	5,08	1.60E-05
GO:0071826	ribonucleoprotein complex subunit organization	49	5,07	6.61E-14

Suppl. Table 3. GO analysis for significantly enriched “Biological processes” (for all identified EV proteins).

Suppl. Table 4. GO analysis for “Biological processes” (all supernatant proteins)

Term ID	Description	Number	Fold enrichment	p value
GO:0006098	pentose-phosphate shunt	7	22,37	3.71E-03
GO:0006740	NADPH regeneration	7	20,14	6.12E-03
GO:0046479	glycosphingolipid catabolic process	8	17,70	1.94E-03
GO:0098761	cellular response to interleukin-7	10	16,92	7.80E-05
GO:0098760	response to interleukin-7	10	16,92	7.80E-05
GO:0019377	glycolipid catabolic process	8	16,44	2.96E-03
GO:0043094	cellular metabolic compound salvage	9	14,38	1.27E-03
GO:0009067	aspartate family amino acid biosynthetic process	10	14,38	2.54E-04
GO:0046514	ceramide catabolic process	9	13,63	1.82E-03
GO:0000097	sulfur amino acid biosynthetic process	7	13,42	4.63E-02
GO:0051156	glucose 6-phosphate metabolic process	8	12,11	1.77E-02
GO:0002183	cytoplasmic translational initiation	8	10,96	3.22E-02
GO:1901658	glycosyl compound catabolic process	9	10,36	1.15E-02
GO:1990748	cellular detoxification	10	10,27	3.13E-03
GO:0009066	aspartate family amino acid metabolic process	13	10,11	6.35E-05
GO:0006418	tRNA aminoacylation for protein translation	14	10,07	1.74E-05
GO:0030149	sphingolipid catabolic process	10	9,59	5.28E-03
GO:0006096	glycolytic process	15	9,38	1.03E-05
GO:0043039	tRNA aminoacylation	14	9,37	3.76E-05
GO:0046364	monosaccharide biosynthetic process	11	9,31	1.84E-03
GO:0006757	ATP generation from ADP	15	9,18	1.32E-05
GO:0043038	amino acid activation	14	9,15	4.80E-05
GO:1901607	alpha-amino acid biosynthetic process	17	9,06	1.27E-06
GO:0045454	cell redox homeostasis	20	8,85	4.25E-08
GO:0006165	nucleoside diphosphate phosphorylation	18	8,63	7.05E-07
GO:0016052	carbohydrate catabolic process	26	8,60	4.75E-11
GO:0046466	membrane lipid catabolic process	10	8,46	1.37E-02
GO:0008652	cellular amino acid biosynthetic process	17	8,43	3.20E-06
GO:0046939	nucleotide phosphorylation	18	8,22	1.38E-06
GO:0097237	cellular response to toxic substance	10	8,22	1.71E-02
GO:0046031	ADP metabolic process	15	8,14	5.21E-05
GO:0006413	translational initiation	15	7,85	7.96E-05
GO:0006749	glutathione metabolic process	16	7,80	2.73E-05
GO:0006081	cellular aldehyde metabolic process	14	7,60	3.50E-04
GO:0042743	hydrogen peroxide metabolic process	11	7,53	1.08E-02
GO:0002181	cytoplasmic translation	17	7,52	1.40E-05
GO:0009179	purine ribonucleoside diphosphate metabolic process	15	7,44	1.46E-04
GO:0009135	purine nucleoside diphosphate metabolic process	15	7,44	1.46E-04
GO:0006090	pyruvate metabolic process	18	7,40	5.85E-06
GO:0098754	detoxification	11	7,36	1.32E-02
GO:0009185	ribonucleoside diphosphate metabolic process	16	7,31	6.07E-05
GO:0061077	chaperone-mediated protein folding	13	7,19	1.85E-03
GO:0006457	protein folding	37	7,10	1.79E-14
GO:0042307	positive regulation of protein import into nucleus	12	6,64	1.13E-02
GO:0009132	nucleoside diphosphate metabolic process	18	6,47	3.64E-05
GO:1904591	positive regulation of protein import	13	6,45	5.43E-03
GO:1900024	regulation of substrate adhesion-dependent cell spreading	12	6,39	1.59E-02
GO:0016051	carbohydrate biosynthetic process	21	6,10	4.74E-06
GO:0019318	hexose metabolic process	26	5,98	6.14E-08
GO:0005996	monosaccharide metabolic process	31	5,75	1.45E-09
GO:0044272	sulfur compound biosynthetic process	15	5,60	3.64E-03
GO:0046824	positive regulation of nucleocytoplasmic transport	14	5,59	9.00E-03
GO:1901657	glycosyl compound metabolic process	19	5,58	1.13E-04
GO:1901136	carbohydrate derivative catabolic process	24	5,52	1.67E-06
GO:0006520	cellular amino acid metabolic process	44	5,30	1.68E-13
GO:1900182	positive regulation of protein localization to nucleus	16	5,23	3.42E-03
GO:0006518	peptide metabolic process	79	5,10	1.33E-25
GO:0006412	translation	53	5,00	7.66E-16

Suppl. Table 4. GO analysis for significantly enriched “Biological processes” (for all identified proteins in EV-depleted cell culture media supernatants).

Suppl. Table 5. List of proteins found only in normoxia EV samples

Accession	Description	Abundances
Q69ZR2	E3 ubiquitin-protein ligase hectd1	345751,5683
Q9JJA4	ribosome biogenesis protein WDR12	430559,5476
Q9D0R8	Protein LSM12 homolog	494949,7815
Q9CW03	Structural maintenance of chromosomes protein 3	568915,5335
Q8VC12	urocanate hydratase	592175,3499
Q8R1A4	Dedicator of cytokinesis protein 7	611802,2361
B1AY13	Ubiquitin carboxyl-terminal hydrolase 24	617469,6283
Q9D071	MMS19 nucleotide excision repair protein homolog	631098,6918
Q8BWU5	Probable tRNA N6-adenosine threonylcarbamoyltransferase	640831,4697
Q8BNV1	tRNA (uracil-5-)-methyltransferase homolog A	653299,3396
O54907	Tumor necrosis factor ligand superfamily member 12	662076,0115
Q6ZQ88	Lysine-specific histone demethylase 1A	692566,0832
P97467	Peptidyl-glycine alpha-amidating monooxygenase	694690,0752
Q8CE96	tRNA (adenine(58)-N(1))-methyltransferase non-catalytic subunit TRM6	700629,2941
Q9D906	Ubiquitin-like modifier-activating enzyme ATG7	712674,2552
Q8VEJ4	Notchless protein homolog 1	721067,9433
Q6PDQ2	Chromodomain-helicase-DNA-binding protein 4	734439,4163
Q8CIH5	1-phosphatidylinositol 4,5-bisphosphate phosphodiesterase gamma-2	768090,4696
O08749	Dihydrolipoyl dehydrogenase, mitochondrial	775735,9248
Q62077	1-phosphatidylinositol 4,5-bisphosphate phosphodiesterase gamma-1	791941,0477
Q8R480	nuclear pore complex protein Nup85	795167,4738
O88796	ribonuclease P protein subunit p30	810029,8639
Q3TCJ1	BRISC complex subunit Abraxas 2	864063,7225
P70388	DNA repair protein Rad50	873863,411
Q9D1C8	vacuolar protein sorting-associated protein 28 homolog	874458,1126
P13439	uridine 5'-monophosphate synthase	897418,859
Q3UMB9	WASH complex subunit 4	915210,1615
P62307	Small nuclear ribonucleoprotein F	922214,2877
Q8R1U1	Conserved oligomeric Golgi complex subunit 4	928436,0008
O08582	GTP-binding protein 1	930578,0361
Q8R242-1	Di-N-acetylchitobiase	946981,989
P08775	DNA-directed RNA polymerase II subunit RPB1	956588,0585
Q9ESJ0	Exportin-4	971199,4497
O35130	Ribosomal RNA small subunit methyltransferase Nep1	982066,2092
Q61081	Hsp90 co-chaperone Cdc37	988929,3605
P97470	Serine/threonine-protein phosphatase 4 catalytic subunit	990864,5736
P32233	developmentally-regulated GTP-binding protein 1	991469,9366
P70261	paladin	1001693,784
P35550	rRNA 2'-O-methyltransferase fibrillarin	1004935,697
Q91VH2	Sorting nexin-9	1010360,52
Q8C2E7	WASH complex subunit 5	1037269,345
Q8VHK9	ATP-dependent RNA helicase DHX36	1039481,402
Q7TQK5	Coiled-coil domain-containing protein 93	1066084,071
Q99J45	Nuclear receptor-binding protein	1073754,129
Q9JKY0	CCR4-NOT transcription complex subunit 9	1074399,683
Q8C3Y4	Kinetochore-associated protein 1	1076129,761
Q60715	prolyl 4-hydroxylase subunit alpha-1	1079584,286
Q3UVG3	Protein FAM91A1	1092808,177
Q8BMF4	Dihydrolipoyllysine-residue acetyltransferase component of pyruvate dehydrogenase complex, mitochondrial	1095597,998

Accession	Description	Abundances
Q9D1Q6	Endoplasmic reticulum resident protein 44	1109475,761
Q8CGK3	Lon protease homolog, mitochondrial	1134475,209
P40936	Indolethylamine N-methyltransferase	1152660,321
Q6PE01	U5 small nuclear ribonucleoprotein 40 kDa protein	1156771,846
Q91WM3	U3 small nucleolar RNA-interacting protein 2	1167374,54
Q3TCH7	cullin-4A	1182636,304
Q99LC2	Cleavage stimulation factor subunit 1	1221212,357
P97855	Ras GTPase-activating protein-binding protein 1	1223021,437
Q8C7R4	Ubiquitin-like modifier-activating enzyme 6	1233081,859
Q9D8N2	Protein FAM45A	1250772,69
Q8R1F1	Niban-like protein 1	1258574,79
O35343	Importin subunit alpha-3	1265453,449
Q9CZH3	Proteasome assembly chaperone 3	1272630,743
Q8C079	striatin-interacting protein 1	1275511,741
Q80WQ2	Protein VAC14 homolog	1295884,451
Q91YJ2	sorting nexin-4	1314289,698
Q8BX09	Retinoblastoma-binding protein 5	1316741,968
Q8CD92	tetratricopeptide repeat protein 27	1323931,082
Q8CGY8-1	UDP-N-acetylglucosamine--peptide N-acetylglucosaminyltransferase 110 kDa subunit	1338490,522
P62869	Elongin-B	1365505,304
Q6NSR8	Probable aminopeptidase NPEPL1	1371841,17
Q8BLN5	lanosterol synthase	1380305,742
P54276	DNA mismatch repair protein MSH6	1387492,726
P10810	Monocyte differentiation antigen CD14	1400300,95
Q8VCT3	aminopeptidase B	1434240,609
Q99KD5	Protein unc-45 homolog A	1434620,411
Q6Q899	Probable ATP-dependent RNA helicase DDX58	1443733,431
Q921G8	Gamma-tubulin complex component 2	1465181,776
Q8VDP4	Cell cycle and apoptosis regulator protein 2	1478123,746
O09164	Extracellular superoxide dismutase	1482690,551
Q6PDI5-1	Proteasome-associated protein ECM29 homolog	1485926,267
Q8K2Z4-2	Isoform 2 of Condensin complex subunit 1	1486041,744
Q9D0F6	Replication factor C subunit 5	1504351,566
Q9JMH9-6	Isoform 6 of Unconventional myosin-XVIIIa	1510649,399
O08759	Ubiquitin-protein ligase E3A	1513184,501
Q8BJ71-1	Nuclear pore complex protein Nup93	1525087,21
Q8C6G8	WD repeat-containing protein 26	1534421,159
Q9QXB9	Developmentally-regulated GTP-binding protein 2	1563336,886
Q8N7N5-1	DDB1- and CUL4-associated factor 8	1587601,055
Q63844	mitogen-activated protein kinase 3	1597915,442
Q99LI7	Cleavage stimulation factor subunit 3	1600013,261
Q99J77	sialic acid synthase	1620246,679
Q9JKV1	Proteasomal ubiquitin receptor ADRM1	1628113,533
Q80UW8	DNA-directed RNA polymerases I, II, and III subunit RPABC1	1632721,018
Q99LC8	Translation initiation factor eIF-2B subunit alpha	1641682,328
O08677-2	Isoform LMW of Kininogen-1	1664108,526
B1AVZ0	Uracil phosphoribosyltransferase homolog	1671245,25
O35345	Importin subunit alpha-7	1738992,589
Q924C1	exportin-5	1785151,519
Q8CFI7	DNA-directed RNA polymerase II subunit RPB2	1795450,85

Accession	Description	Abundances
Q8VI75	Importin-4	1819930,313
Q9JI33	Netrin-4	1838152,239
P54823	Probable ATP-dependent RNA helicase DDX6	1838911,736
P27641	X-ray repair cross-complementing protein 5	1880473,789
P83887	tubulin gamma-1 chain	1883790,684
Q9D2R0	acetoacetyl-CoA synthetase	1901974,84
Q9QXC1	Fetuin-B	1903492,44
Q6PB93-1	Polypeptide N-acetylgalactosaminyltransferase 2	1904192,393
E9Q4P1	WD repeat and FYVE domain-containing protein 1	1907067,595
Q68FH4	N-acetylgalactosamine kinase	1918893,602
P61967	AP-1 complex subunit sigma-1A	1924531,111
Q9EPK6	nucleotide exchange factor SIL1	1940724,45
Q8BP48	Methionine aminopeptidase 1	1943727,831
P27661	Histone H2AX	1955550,915
Q91ZW3	SWI/SNF-related matrix-associated actin-dependent regulator of chromatin subfamily A member 5	1965777,065
Q9D7M1	Glucose-induced degradation protein 8 homolog	1997595,666
Q6PAR5-4	Isoform 4 of GTPase-activating protein and VPS9 domain-containing protein 1	2021598,412
Q9ER38	Torsin-3A	2030251,592
Q9D0K2	Succinyl-CoA:3-ketoacid coenzyme A transferase 1, mitochondrial	2039027,999
Q01279	epidermal growth factor receptor	2053595,649
Q8K297	Procollagen galactosyltransferase 1	2069964,235
Q99LJ1	tissue alpha-L-fucosidase	2104542,772
P59328	WD repeat and HMG-box DNA-binding protein 1	2141801,038
Q6PEB6	MOB-like protein phocein	2168953,457
Q8VHN8	Tudor-interacting repair regulator protein	2193507
P27090	Transforming growth factor beta-2	2197470,785
Q8BVG4-1	Dipeptidyl peptidase 9	2205756,584
Q9CQI7	U2 small nuclear ribonucleoprotein B''	2283078,086
Q9CZU3	Superkiller viralicidic activity 2-like 2	2297452,546
Q9CXK8	60S ribosome subunit biogenesis protein NIP7 homolog	2351200,09
Q8K1R7	Serine/threonine-protein kinase Nek9	2376192,64
Q9EP82	tRNA (guanine-N(7)-)-methyltransferase non-catalytic subunit WDR4	2378612,933
Q91ZJ9-1	Hyaluronidase-1	2413907,815
P51807	Dynein light chain Tctex-type 1	2421018,432
Q80UM7	Mannosyl-oligosaccharide glucosidase	2441173,34
Q9JK38	glucosamine 6-phosphate N-acetyltransferase	2471927,901
P37040	NADPH--cytochrome P450 reductase	2521584,886
Q8K2V6-2	Isoform 2 of Importin-11	2528716,406
Q99LE6	ATP-binding cassette sub-family F member 2	2559754,087
Q80TM9-1	Nischarin	2572014,912
Q7TMY8	E3 ubiquitin-protein ligase HUWE1	2588124,763
Q9QXP7	Complement C1q tumor necrosis factor-related protein 1	2687686,3
P52432	DNA-directed RNA polymerases I and III subunit RPAC1	2773972,649
Q8VCF1-3	Isoform 3 of Soluble calcium-activated nucleotidase 1	2815081,864
Q9Z1Z0-1	General vesicular transport factor p115	2868011,127
P68369	tubulin alpha-1A chain	2870153,992
P68181-4	Isoform 4 of cAMP-dependent protein kinase catalytic subunit beta	3043735,061
Q91YL3	Uridine-cytidine kinase-like 1	3048515,258
Q9ERF3	WD repeat-containing protein 61	3130361,641
Q6PGN1	Erythroferrone	3150167,468

Accession	Description	Abundances
P99027	60S acidic ribosomal protein P2	3217874,494
Q64471	Glutathione S-transferase theta-1	3265685,923
Q9WUM3	Coronin-1B	3351556,124
P54751	CMP-N-acetylneuraminate-beta-galactosamide-alpha-2,3-sialyltransferase 1	3388575,982
Q91WC0	Histone-lysine N-methyltransferase setd3	3518963,381
Q5XJY5	Coatomer subunit delta	3559542,42
Q1HFZ0-1	tRNA (Cytosine(34)-C(5))-methyltransferase	3567261,103
A2AKG8	focadhesin	3613542,715
Q8R1J9	Torsin-2A	3685482,897
P18654	Ribosomal protein S6 kinase alpha-3	3811308,371
Q8VDW0-1	ATP-dependent RNA helicase DDX39A	3941030,739
Q9WV60	Glycogen synthase kinase-3 beta	4039254,306
Q6ZQ08-4	Isoform 4 of CCR4-NOT transcription complex subunit 1	4170488,034
O54950	5'-AMP-activated protein kinase subunit gamma-1	4296635,731
Q3UYV9	Nuclear cap-binding protein subunit 1	4662009,647
O35226-2	Isoform Rpn10B of 26S proteasome non-ATPase regulatory subunit 4	5075922,813
Q6S5C2-2	Isoform 2 of N-acetylglucosamine-1-phosphotransferase subunit gamma	5112843,939
Q9D6X6	Serine protease 23	5151391,424
Q8QZR4	Out at first protein homolog	5189992,701
Q9Z1R2	Large proline-rich protein BAG6	5223247,638
O08573	Galectin-9	5639373,405
O35134	DNA-directed RNA polymerase I subunit RPA1	5809155,944
Q9D2V7	Coronin-7	5809306,229
A2A5R2	Brefeldin A-inhibited guanine nucleotide-exchange protein 2	5924930,958
Q09324	beta-1,3-galactosyl-O-glycosyl-glycoprotein beta-1,6-N-acetylglucosaminyltransferase	5987473,022
Q6IR41	complement C1q tumor necrosis factor-related protein 6	6041974,008
O55106	striatin	6256267,494
Q99L27	GMP reductase 2	6272918,61
Q923T9-1	Calcium/calmodulin-dependent protein kinase type II subunit gamma	6429935,483
Q99JN2-2	Isoform 2 of Kelch-like protein 22	6592184,542
P60335	Poly(RC)-binding protein 1	6999199,14
P59325	Eukaryotic translation initiation factor 5	7183686,835
Q9ES89	Exostosin-like 2	7493645,792
Q922K7	Probable 28S rRNA (cytosine-C(5))-methyltransferase	7763669,53
P47856	glutamine--fructose-6-phosphate aminotransferase	8032540,406
Q9Z0F8	Disintegrin and metalloproteinase domain-containing protein 17	8877042,776
Q9R1Q9	V-type proton ATPase subunit S1	9996429,714
Q9CRT8	Exportin-T	11017346,73
P52293	Importin subunit alpha-1	11116943,38
Q9D4H8	Cullin-2	12050689,44
Q8CG48	structural maintenance of chromosomes protein 2	13241474,55
P39054-1	Dynamin-2	13526111,42
O35988	syndecan-4	14440333,61
Q9CQ01	Ribonuclease T2	15056108,6
Q8BPB5	EGF-containing fibulin-like extracellular matrix protein 1	15514790,16
Q9EPU0-1	Regulator of nonsense transcripts 1	22972963,94
Q922F4	Tubulin beta-6 chain	27430681,18

Suppl. Table 5. List of proteins found only in normoxia EV samples (cf. Suppl. Table 1).

Suppl. Table 6. List of proteins found only in hypoxia EV samples

Accession	Description	Abundances
Q9CQ80	Vacuolar protein-sorting-associated protein 25	533374,4727
Q9CWZ7	Gamma-soluble NSF attachment protein	631989,0484
Q8R0J7	Vacuolar protein sorting-associated protein 37B	670889,3921
P63321	Ras-related protein Ral-A	766052,6296
G5E829	Plasma membrane calcium-transporting ATPase 1	772747,9559
Q61072	disintegrin and metalloproteinase domain-containing protein 9	788437,2099
F6ZDS4	Nucleoprotein TPR	863541,5372
Q9Z0L0	Trophoblast glycoprotein	916511,1117
Q8CIN4	Serine/threonine-protein kinase PAK 2	923054,3287
Q9CQX5	Claudin domain-containing protein 1	923602,6171
Q99L13	3-hydroxyisobutyrate dehydrogenase, mitochondrial	1026677,855
Q922J3-1	CAP-Gly domain-containing linker protein 1	1030447,968
Q80W68	Kin of IRRE-like protein 1	1243999,756
P08556	GTPase NRas	1280374,784
Q99P31	Hsp70-binding protein 1	1324826,975
Q9D8T2	Gasdermin-D	1349422,975
P32507	Nectin-2	1353582,485
P62746	Rho-related GTP-binding protein RhoB	1360597,391
Q91W53	Golgin subfamily A member 7	1368898,726
Q6PB44-1	Tyrosine-protein phosphatase non-receptor type 23	1377079,195
Q03963	interferon-induced, double-stranded RNA-activated protein kinase	1399402,927
Q9D358	Low molecular weight phosphotyrosine protein phosphatase	1438416,085
P97797	tyrosine-protein phosphatase non-receptor type substrate 1	1445509,783
Q9JJU8	SH3 domain-binding glutamic acid-rich-like protein	1515378,717
Q8K135-2	Isoform 2 of Dyslexia-associated protein KIAA0319-like protein	1564716,106
P49769	Presenilin-1	1585311,322
P60904	DnaJ homolog subfamily C member 5	1589417,774
Q9D8C4	Interferon-induced 35 kDa protein homolog	1647963,089
Q3UH60-1	Disco-interacting protein 2 homolog B	1689681,686
Q9JI75	Ribosyldihydronicotinamide dehydrogenase	1730691,509
O88811-1	Signal transducing adapter molecule 2	1790043,515
Q9Z2M7	Phosphomannomutase 2	1948741,464
P06869	Urokinase-type plasminogen activator	1971636,399
Q9CPV4-1	Glyoxalase domain-containing protein 4	2023539,501
Q9D1G1	ras-related protein Rab-1B	2072201,322
O54951	Semaphorin-6B	2073762,03
Q9Z1G3	V-type proton ATPase subunit C 1	2131825,636
Q9JLF6	Protein-glutamine gamma-glutamyltransferase K	2151460,276
Q8BZW8	NHL repeat-containing protein 2	2299367,504
Q9JHZ2	Progressive ankylosis protein	2358212,629
P10833	Ras-related protein R-Ras	2383875,784
P10711	Transcription elongation factor A protein 1	2401048,24
P27512	Tumor necrosis factor receptor superfamily member 5	2457417,933
Q64433	10 kDa heat shock protein, mitochondrial	2516133,347
P23506-2	Isoform 2 of Protein-L-isoaspartate(D-aspartate) O-methyltransferase	2599090,387
P21460	Cystatin-C	2871251,644
O35054	Claudin-4	2892103,347
Q61738-3	Isoform Alpha-7X1B of Integrin alpha-7	2898614,987
P62748	Hippocalcin-like protein 1	2973712,55
P61021	Ras-related protein Rab-5B	3046195,008

Accession	Description	Abundances
P15702	Leukosialin	3199332,656
Q8K157	aldose 1-epimerase	3201022,153
Q6X893-2	Isoform 2 of Choline transporter-like protein 1	3250986,008
Q9CQW1	Synaptobrevin homolog YKT6	3378057,705
Q3UHH2	Solute carrier family 22 member 23	3409634,415
P62835	ras-related protein Rap-1A	3431813,768
Q99KB8	Hydroxyacylglutathione hydrolase, mitochondrial	3603720,069
P45591	Cofilin-2	3610547,197
Q3UHK6-3	Isoform 3 of Teneurin-4	3872667,271
Q9CQV6	Microtubule-associated proteins 1A/1B light chain 3B	3903148,166
Q91YI0	argininosuccinate lyase	3928134,202
Q9CQD1	Ras-related protein Rab-5A	4146223,703
O35682	myeloid-associated differentiation marker	4157729,768
P08228	Superoxide dismutase	4276591,698
Q09143	High affinity cationic amino acid transporter 1	4804926,124
Q6P069-1	Sorcin	5012726,38
P13597-1	Intercellular adhesion molecule 1	5108041,667
Q9WTP6-1	Adenylate kinase 2, mitochondrial	5310297,875
O88792	Junctional adhesion molecule A	5824759,384
P14901	heme oxygenase 1	6201874,562
Q9CQD4	Charged multivesicular body protein 1b-2	6214724,318
Q8BY89-1	Choline transporter-like protein 2	6304006,098
Q8BHL4	Retinoic acid-induced protein 3	6424755,042
O70404	vesicle-associated membrane protein 8	6859418,208
Q9EQX4	allograft inflammatory factor 1-like	7298200,844
P97372	proteasome activator complex subunit 2	7443928,513
Q91Z53	Glyoxylate reductase/hydroxypyruvate reductase	7491127,936
Q8BGK6-1	Y+L amino acid transporter 2	8539174,546
P97792-1	Coxsackievirus and adenovirus receptor homolog	8591552,856
O35874	neutral amino acid transporter A	8768336,497
Q63961	Endoglin	8851079,379
Q6P9J9	Anoctamin-6	10047934,5
P57716	Nicastrin	11120648,07
P62897	Cytochrome c, somatic	11762164,4
P31786	acyl-CoA-binding protein	11913530,48
P97371	Proteasome activator complex subunit 1	11930112,62
P53994	Ras-related protein Rab-2A	15595526,16
P35846	Folate receptor alpha	16371639,67
O35316	Sodium- and chloride-dependent taurine transporter	17474191,68
Q8C0Z1	Protein FAM234A	18909542,32
Q61735-2	Isoform 2 of Leukocyte surface antigen CD47	19632644,67
Q8CI85	Carbonic anhydrase 12	20457001,78
P28571-1	Isoform GlyT-1A of Sodium- and chloride-dependent glycine transporter 1	21346356,8
Q69ZN7-1	Myoferlin	25476556,61
Q62011	Podoplanin	28275794,2
P15379-14	CD44 antigen	30428609,41
P97370	sodium/potassium-transporting ATPase subunit beta-3	69848978,36
Q60634-1	Flotillin-2	80021450,91

Suppl. Table 6. List of proteins found only in hypoxia EV samples (cf. Suppl. Table 1).
